# Pivotal Roles for pH, Lactate, and Lactate-Utilizing Bacteria in the Stability of a Human Colonic Microbial Ecosystem

**DOI:** 10.1128/mSystems.00645-20

**Published:** 2020-09-08

**Authors:** Shui Ping Wang, Luis A. Rubio, Sylvia H. Duncan, Gillian E. Donachie, Grietje Holtrop, Galiana Lo, Freda M. Farquharson, Josef Wagner, Julian Parkhill, Petra Louis, Alan W. Walker, Harry J. Flint

**Affiliations:** a College of Animal Science, Southwest University, Chongqing, People’s Republic of China; b Physiology and Biochemistry of Animal Nutrition (EEZ, CSIC), Granada, Spain; c Gut Health Group, Rowett Institute, University of Aberdeen, Foresterhill, Aberdeen, Scotland, UK; d Biomathematics and Statistics Scotland (BioSS), Aberdeen, Scotland, UK; e Pathogen Genomics Group, Wellcome Sanger Institute, Hinxton, Cambridgeshire, UK; f Department of Veterinary Medicine, University of Cambridge, Cambridge, UK; Teagasc Food Research Centre

**Keywords:** lactate, lactate-utilizing bacteria, pH, short-chain fatty acids, mathematical modeling

## Abstract

Lactate can be produced by many gut bacteria, but in adults its accumulation in the colon is often an indicator of microbiota perturbation. Using continuous culture anaerobic fermentor systems, we found that lactate concentrations remained low in communities of human colonic bacteria maintained at pH 6.5, even when dl-lactate was infused at 10 or 20 mM. In contrast, lower pH (5.5) led to periodic lactate accumulation following lactate infusion in three fecal microbial communities examined. Lactate accumulation was concomitant with greatly reduced butyrate and propionate production and major shifts in microbiota composition, with *Bacteroidetes* and anaerobic *Firmicutes* being replaced by *Actinobacteria*, lactobacilli, and *Proteobacteria*. Pure-culture experiments confirmed that *Bacteroides* and *Firmicutes* isolates were susceptible to growth inhibition by relevant concentrations of lactate and acetate, whereas the lactate-producer Bifidobacterium adolescentis was resistant. To investigate system behavior further, we used a mathematical model (microPop) based on 10 microbial functional groups. By incorporating differential growth inhibition, our model reproduced the chaotic behavior of the system, including the potential for lactate infusion both to promote and to rescue the perturbed system. The modeling revealed that system behavior is critically dependent on the proportion of the community able to convert lactate into butyrate or propionate. Communities with low numbers of lactate-utilizing bacteria are inherently less stable and more prone to lactate-induced perturbations. These findings can help us to understand the consequences of interindividual microbiota variation for dietary responses and microbiota changes associated with disease states.

**IMPORTANCE** Lactate is formed by many species of colonic bacteria, and can accumulate to high levels in the colons of inflammatory bowel disease subjects. Conversely, in healthy colons lactate is metabolized by lactate-utilizing species to the short-chain fatty acids butyrate and propionate, which are beneficial for the host. Here, we investigated the impact of continuous lactate infusions (up to 20 mM) at two pH values (6.5 and 5.5) on human colonic microbiota responsiveness and metabolic outputs. At pH 5.5 in particular, lactate tended to accumulate in tandem with decreases in butyrate and propionate and with corresponding changes in microbial composition. Moreover, microbial communities with low numbers of lactate-utilizing bacteria were inherently less stable and therefore more prone to lactate-induced perturbations. These investigations provide clear evidence of the important role these lactate utilizers may play in health maintenance. These should therefore be considered as potential new therapeutic probiotics to combat microbiota perturbations.

## Abstract (precis)

Lactate is formed by many species of colonic bacteria, and can accumulate to high levels in the colons of inflammatory bowel disease subjects. Conversely, in healthy colons lactate is metabolized by lactate-utilizing species to the short-chain fatty acids butyrate and propionate, which are beneficial for the host. Here, we investigated the impact of continuous lactate infusions (up to 20 mM) at two pH values (6.5 and 5.5) on human colonic microbiota responsiveness and metabolic outputs. At pH 5.5 in particular, lactate tended to accumulate in tandem with decreases in butyrate and propionate and with corresponding changes in microbial composition. Moreover, microbial communities with low numbers of lactate-utilizing bacteria were inherently less stable and therefore more prone to lactate-induced perturbations. These investigations provide clear evidence of the important role these lactate utilizers may play in health maintenance. These should therefore be considered as potential new therapeutic probiotics to combat microbiota perturbations.

## INTRODUCTION

The microbial community of the large intestine is known to differ greatly in species composition between individuals. Nevertheless, in healthy individuals the community displays a considerable degree of stability in species composition and metabolite outputs from day to day (1). Meanwhile, interindividual variation is generally more pronounced at the level of individual species than at the level of phyla or broad functional groups. This makes it possible for us to ask some generic questions about the response of the human intestinal microbiota to perturbation. Imposed changes in pH, for example, have been shown previously to cause broad shifts in metabolite outputs and species composition in human colonic communities maintained in *in vitro* continuous culture (2, 3). *In vivo*, the pH of the intestinal lumen is largely determined by the concentrations of short-chain acids being produced by the fermentation of dietary fiber (4). This means that the activities of the gut microbiota have the potential to feed back on its behavior and species composition by altering the gut environment. Potentially, this applies not only to pH but also to any effects of individual fermentation acids, or indeed of other metabolic products, on microbial growth.

In this study, we focus on the potential impact of changes in lactate concentration and lactate supply upon the colonic microbial community at two different pH values. Lactic acid is a product of both mammalian metabolism and of gut bacterial fermentation of carbohydrates under anaerobic conditions. Lactate enters the gut from the bloodstream but is also produced by the gut microbiota in both the small intestine, where the microbiota is typically dominated by lactic acid-producing bacteria, and in the colon, which harbors many bacterial species with the potential to produce lactate among other fermentation products (5).

Lactate has been shown to inhibit the growth of some pathogenic bacteria, including Escherichia coli (6), and can also reach high concentrations in the gut of healthy infants, reflecting the dominance of l-lactate-producing bifidobacteria (7, 8). However, it can also exert an array of deleterious effects, in particular due to its potential to drive down gut pH as a result of its low pKa, which can lead to changes in the microbiota and to acidosis in the adult colon (9). It can also be used as a growth substrate by sulfate-reducing gut bacteria (10) and so has the potential to promote the formation of toxic concentrations of hydrogen sulfide, which has been linked to colonic disease (11). Lactate has also been shown to accumulate in the colon in progressively greater amounts under conditions of increasingly severe colitis (12, 13) and in the small intestine microbially produced d-lactate is a neurotoxin that can prove fatal in cases of short-bowel syndrome (14). Recent work has also revealed that lactate may promote the growth of certain pathogens such as Salmonella enterica serovar Typhimurium, which can utilize lactate and therefore benefits from a perturbed gut environment with lactate accumulation. Indeed, *Salmonella* infection is reported to suppress formation of the beneficial short-chain fatty acid (SCFA) butyrate and to increase the production of l-lactate from glucose by the colonic epithelium (15). Lactate can also enhance immune evasion by the fungal pathogen Candida albicans by triggering masking of its cell wall antigens (16).

Rather little is known about the impact of lactate influx, arising either from the small intestine or from host tissues, upon the colonic microbiota. There is scant information on lactic acid concentration in the different human intestinal compartments under normal conditions. However, in pigs, small intestinal lactic acid concentrations have been reported to be 50 to 100 mM, while the total cumulative concentration of other SCFAs, such as acetate, propionate, and butyrate, is just 5 mM. In the large intestine, however, those proportions are reversed (17–19). Importantly, lactate does not appear to accumulate in the colon of healthy adult humans either, even though it can be produced by many gut bacteria. Indeed, concentrations of just 5 to 7 mM lactate have been reported in the ascending colons of sudden-death victims with around 180 mM total SCFAs (20).

The reason that lactate does not accumulate in the adult human colon under normal health conditions, despite being produced by many gut anaerobes, is that the gut microbiota includes lactate-utilizing bacteria (which we refer to here generically as “LUB”) that can utilize lactate for growth. The activities of these LUB therefore play an important role in determining lactate concentrations (21). Prominent lactate utilizers include particular species of the *Firmicutes* phylum that are able to produce the SCFAs butyrate or propionate from lactate (5, 22). Since these two SCFAs are known to exert a number of positive effects for the host (23), lactate utilization can be considered an indirectly beneficial consequence of lactate production by other members of the gut microbiota. However, it is known that some of the previously described lactate-utilizing species are sensitive to reduced pH, which may help to explain why lactate accumulates under conditions of a mildly acidic pH but not at a more neutral pH (24, 25). Further work is required to better understand the potential roles that lactate utilizers may play in maintaining the healthy adult colon.

Here, we use a combination of experimental and theoretical modeling approaches to explore the role of lactate and lactate utilization in the stability of gut microbial communities. Our experimental approach uses pH-controlled anaerobic continuous cultures inoculated with fecal bacteria and supplied with polysaccharides as growth substrates. We show that LUB within the gut microbiota have a remarkable capacity to consume lactate, thereby stabilizing the system, and that systemic pH is a significant driver of this activity. Theoretical modeling broadly reproduced these changes and is consistent with hypotheses regarding (i) the selective inhibition of the two major phyla of commensal gut anaerobes (*Bacteroidetes* and *Firmicutes*) by lactate and (ii) the populations and activities of LUB, which both play key roles in the observed community shifts. Lactate accumulation is shown here to result in perturbed communities that are dominated by *Proteobacteria* and other facultative anaerobes, mirroring changes that have been reported for microbial community perturbations associated with conditions such as diarrhea in humans (26).

## RESULTS

### Impact of fecal inoculum, pH, and lactate infusions on colonic microbiota present in fermentor vessels.

**FIG 1:**
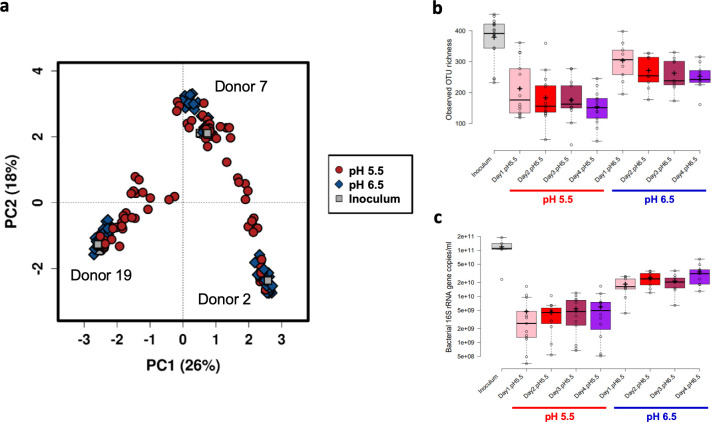
Impact of pH on fecal microbiota composition within continuous culture fermentor vessels. (a) PCA plot showing the impact of fecal donor and pH on overall microbiota clustering patterns. The text adjacent to each main cluster on the plot indicates the fecal donor (also visualized in the accompanying Fig. S1b). Samples held at pH 5.5 were more variable than those held at pH 6.5 and the original inocula samples, which tended to cluster more tightly. (b) Bacterial richness in original fecal inocula samples, and fermentor samples held at pH 5.5 or 6.5 over the 4-day course of the experiments. (c) Log_10_ scale 16S rRNA gene counts/ml, as assessed by qPCR, in original fecal inocula samples, and in fermentor samples held at pH 5.5 or 6.5 over the 4-day course of the experiments. For panels b and c, the center lines show the medians; the box limits indicate the 25th and 75th percentiles as determined by R software; the whiskers extend 1.5 times the interquartile range from the 25th and 75th percentiles, with outliers represented by dots; crosses represent sample means; and data points are plotted as open circles.

pH-controlled continuous flow fermentor systems, at either pH 5.5 or 6.5, mimicking the approximate pH of the proximal and distal colons, respectively (27), were supplied with culture medium containing 0.74% mixed polysaccharides and 0.2% peptides, and were inoculated with fecal microbiota (as detailed in Materials and Methods). Fermentors held at both pHs were continuously infused with either 20 mM (“high”), 10 mM (“medium”), or zero lactate (which we term “low” rather than “zero” since there is always a small amount of lactate present in fermentors due to its continual production by some members of the fermentor microbial community), in order to monitor the overall impact on the microbial community structure. The experiment was repeated with three different fecal microbiota donors. The overall microbiota clustering patterns, based upon bacterial 16S rRNA gene sequence analysis, showed that fecal donor was the largest driver of clustering patterns, but that pH and lactate infusions were also important contributors (for all three variables *P* < 0.001, using the parsimony test in mothur) (see Fig. S1a in the supplemental material). In agreement, principal-component analysis (PCA) revealed that, although samples clustered by donor (Fig. S1b), the data points derived from samples at pH 5.5 were more widely distributed throughout the plot, indicating that the lower pH had a greater destabilizing impact on the microbiota (Fig. 1a). Furthermore, both observed bacterial operational taxonomic unit (OTU) richness (Fig. 1b) and overall bacterial loads, as assessed by qPCR (Fig. 1c), were consistently lower in the fermentor vessels maintained at pH 5.5 than both the original inocula samples and fermentor vessels at pH 6.5. Similar initial reductions in OTU diversity have been reported previously (28) and are assumed to result mainly from the more limited substrate diversity and constant environmental conditions compared to the situation *in vivo*. Dead cells derived from higher up in the gastrointestinal tract, which can still be detected in fecal inocula using DNA-based methods, may also be a contributing factor, as these will be rapidly washed out of the fermentor systems as the growth medium is continuously replenished. Our results here show that the prevailing pH of the system is also crucially important, with the lower pH clearly limiting the growth of many species.

10.1128/mSystems.00645-20.1 FIG S1 (a) Dendrogram, based on Bray-Curtis dissimilarity at the OTU-level, showing overall microbiota clustering patterns. Samples collected from fermentor vessels at time points 0 h and 8 h were grouped together with those from the initial fecal inocula as the microbial communities had not yet had sufficient time to grow and adapt to the conditions inside the fermentors. As such, they were still strongly reflective of the compositions in the original inocula. Inner ring indicates which fecal donor the samples originated from, middle ring indicates the impact of pH on the overall clustering patterns, and outer ring shows the amount of lactate that was added to the fermentor systems (“L” = zero, “M” = 10 mM, “H” = 20 mM). (b) Principal component analysis (PCA) plot showing the impact of fecal donor on overall microbiota clustering patterns. The same plot, colored instead to visualize the impact of pH, is shown in Fig. 1a in the main text. Download FIG S1, TIF file, 1.0 MB. Copyright © 2020 Wang et al. 2020 Wang et al. This content is distributed under the terms of the Creative Commons Attribution 4.0 International license.

Having established the importance of pH, we then explored the dynamics within fermentor systems maintained at the two different pHs separately.

### Stability of the microbial community in continuous culture at pH 6.5.

**FIG 2:**
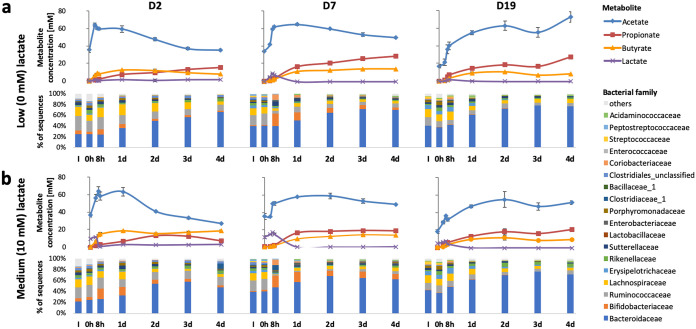
Changes in short-chain fatty acid concentrations and relative abundance of bacterial families in continuous flow fermentor communities derived from three fecal donors (D2, D7, and D19) controlled at pH 6.5. (a) No infusion of lactate; (b) continual infusion of 10 mM dl-lactate.

The time course of changes in community composition and metabolism following inoculation at pH 6.5 are shown in Fig. 2 and Fig. S2a. Short-chain fatty acid concentrations (ranging from 65 to 110 mM) between two and 4 days were typical of fecal samples from healthy adults (4). Sequencing analysis at the family-level revealed a steady increase in *Bacteroidaceae*, which accounted for 70% of 16S rRNA gene sequences after 4 days of incubation at pH 6.5. The dominance of *Bacteroidaceae* when soluble polysaccharide substrates are supplied at pH 6.5 agrees closely with fermentor-based experiments reported previously (2, 3, 29, 30). Full data, including proportional abundance and taxonomic classification of individual OTUs, are given in Table S1 in the supplemental material.

10.1128/mSystems.00645-20.2 FIG S2 Changes in short-chain fatty acid concentrations and relative abundance of bacterial families in continuous flow fermentor communities derived from three fecal donors (D2, D7, and D19) with dl-lactate continually infused at 20 mM. (a) pH controlled at 6.5; (b) pH controlled at 5.5. Download FIG S2, TIF file, 0.3 MB. Copyright © 2020 Wang et al. 2020 Wang et al. This content is distributed under the terms of the Creative Commons Attribution 4.0 International license.

10.1128/mSystems.00645-20.8 TABLE S1 Full 16S rRNA gene sequence data showing the distribution of individual OTUs per sample, and their taxonomic classifications, as well as individual sample ENA accession number information. Download Table S1, XLSX file, 2.0 MB. Copyright © 2020 Wang et al. 2020 Wang et al. This content is distributed under the terms of the Creative Commons Attribution 4.0 International license.

Each of these experiments involved two additional, parallel, vessels that received identical fecal inocula and were continuously infused with medium containing the same mix of polysaccharides, but which also received in addition a continuous infusion of either 10 mM (Fig. 2b) or 20 mM dl-lactate (Fig. S2a). Lactate infusion did not greatly alter either the major metabolite concentrations or microbial community composition from 48 to 96 h. In addition, LEfSe analysis showed that, overall, no bacterial families, genera or OTUs were significantly associated with any of the three lactate infusion levels at pH 6.5 after correcting for the false discovery rate (FDR) (Table S2a). In some cases, lactate accumulated before 48 h, with a proportionally increased bifidobacterial population presumably contributing more lactate as a product of polysaccharide fermentation (Fig. 2; see also Fig. S2a), but lactate concentrations remained low (below 4 mM) after 48 h, indicating efficient lactate utilization by other species within the community.

10.1128/mSystems.00645-20.9 TABLE S2 (a) LEfSe results for biomarkers of high, medium, and low levels of lactate at pH 6.5. (b) LEfSe results for biomarkers of high, medium and low levels of lactate at pH 5.5. Download Table S2, XLSX file, 0.1 MB. Copyright © 2020 Wang et al. 2020 Wang et al. This content is distributed under the terms of the Creative Commons Attribution 4.0 International license.

### Instability of the microbial community in continuous culture at pH 5.5.

**FIG 3:**
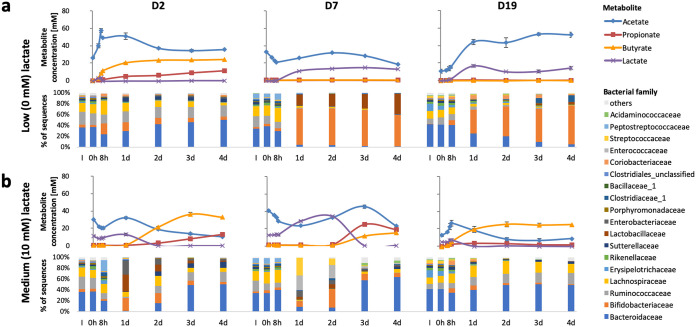
Changes in short-chain fatty acid concentrations and relative abundance of bacterial families in continuous flow fermentor communities derived from three fecal donors (D2, D7, and D19) controlled at pH 5.5. (a) No infusion of lactate; (b) continual infusion of 10 mM dl-lactate.

When the same experiments were run for the D2 microbiota with the pH held at 5.5, the proportion of *Bacteroides* was lower than that observed at pH 6.5, and there was instead a greater proportion of butyrate-producing *Firmicutes* species. This was accompanied by higher butyrate levels than at pH 6.5 in the presence of up to 10 mM lactate (Fig. 3). These observations were in agreement with previous reports showing that *Bacteroides* spp. are less dominant, and that the overall butyrate production is enhanced, in fermentor systems held at the lower pH (2). For the other two donors (D7 and D19), however, pH 5.5 with zero lactate infusion caused a dramatic shift to the production of acetate and lactate, with little or no propionate or butyrate formed. This corresponded to a greatly increased proportional representation of lactate-producing bifidobacteria (and lactobacilli in donor D7 only), within the community from days 1 to 4 (Fig. 3a). Remarkably, infusion of 10 mM lactate at pH 5.5 resulted in lower lactate concentrations at day 4 in these two donors (Fig. 3b), together with restoration of a community profile closer to that of the inoculum. Partial restoration of propionate and butyrate formation at day 4 was also seen with infusion of 20 mM lactate for D19 and in an extended repeat fermentor run for D2 (see Fig. S2b and Fig. S3a). In the case of D7 with 20 mM lactate infusion, metabolic activity appeared to be limited to the partial conversion of lactate to acetate, suggesting that there was little fermentation of the input polysaccharides to SCFA, but perhaps with possible conversion of one stereoisomer of the input DL-lactate to acetate (Fig. S3b). There is evidence to suggest that some gut anaerobes might be able to convert lactate to acetate (see, for example, reference 31), but the taxonomic groups involved are not fully characterized, and we were therefore not able to definitively assign this activity to specific taxa in our fermentor experiments.

10.1128/mSystems.00645-20.3 FIG S3 Outcome of replicate fermentor experiments with pH controlled at 5.5. (a) Donor 2, showing an extended repeat run with infusion of 20 mM lactate; (b) donor 7 (0, 10, and 20 mM lactate infusion); (c) donor 19 (0, 10, and 20 mM lactate infusion). Download FIG S3, PDF file, 0.2 MB. Copyright © 2020 Wang et al. 2020 Wang et al. This content is distributed under the terms of the Creative Commons Attribution 4.0 International license.

**FIG 4:**
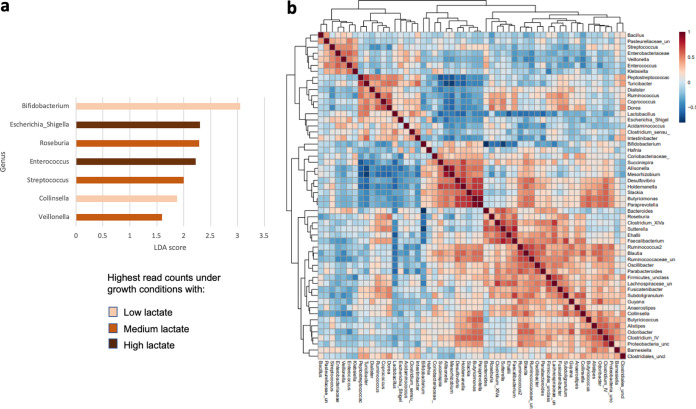
(a) LEfSe results showing the genera that were significantly associated with different lactate infusion levels at pH 5.5. All *P* values were <0.05 after correction for FDR. (b) Spearman correlation network for bacterial genera in the fermentor systems at pH 5.5. Correlations were based on data from all donors, including repeat fermentor runs. In order to focus on the most abundant genera, only reads that occurred more than five times, and which were detected in at least 10% of samples, were included in this comparison. A total of 57 samples, encompassing all pH 5.5 fermentor samples collected after the 24-h time point, were included in both of these comparisons. Samples from the earlier time points were excluded as they more reflect the composition of the initial fecal inocula rather than the communities that had adapted to the conditions inside the fermentors.

Despite the apparently chaotic behavior of the system at pH 5.5, repeats of the D2, D7, and D19 experiments largely gave very similar outcomes (Fig. S3a to c, respectively). These perturbed metabolic profiles were mirrored by 16S rRNA gene sequence-based taxonomic results (Fig. 4). While low levels of lactate at pH 5.5 were correlated with increased proportional abundances of bifidobacteria and *Collinsella* spp. (Fig. 4a; see also Table S2b), infusion of higher levels of lactate were correlated with increased proportional abundances of facultative anaerobes linked to microbiota perturbations such as *Escherichia/Shigella* and *Enterococcus* spp. (Fig. 4a). Furthermore, Spearman correlation analysis indicated that, overall, facultative anaerobes such as *Klebsiella*, *Streptococcus*, *Bacillus*, and *Enterococcus* spp. appeared to be correlated with each other (Fig. 4b, top left-hand cluster on the dendrograms), likely driven by selective pressure of the differing lactate concentrations at the lower pH.

### Absolute bacterial numbers, populations of presumptive lactate-utilizing bacteria, and methanogenic archaea.

**FIG 5:**
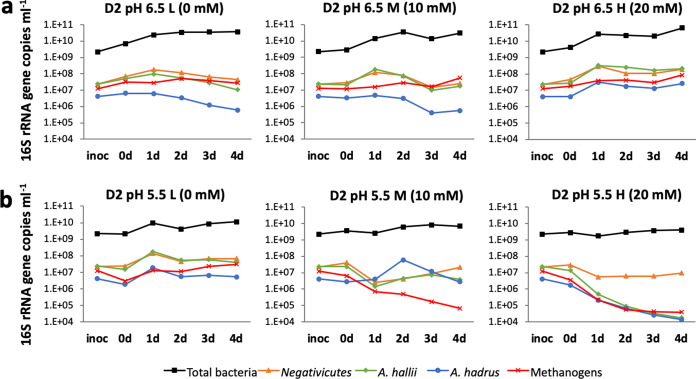
Changes in 16S rRNA gene count estimated by qPCR for three groups of lactate-utilizing *Lachnospiraceae*, total bacteria and methanogenic archaea in fermentors inoculated with D2 microbiota at pH 6.5 (a) and at pH 5.5 (b).

Total bacterial numbers and populations of methanogenic archaea were estimated by qPCR; results from D2 are shown in Fig. 5 and those from donors D7 and D19 in Fig. S4a and b, respectively. Also shown are the populations of bacteria related to *Anaerobutyricum* (formerly *Eubacterium* [32]) *hallii*, Anaerostipes hadrus, and *Negativicutes*, all of which are bacterial lineages that have previously been demonstrated to include lactate-utilizers and which were estimated using group-specific qPCR primer sets. Methanogens were only abundant in fecal samples from D2, but it can be noted that their populations were maintained in the fermentors run at pH 6.5 with 0, 10, or 20 mM lactate infusion. At pH 5.5, however, methanogen populations were only maintained when there was no lactate infusion (Fig. 5).

10.1128/mSystems.00645-20.4 FIG S4 Changes in 16S rRNA gene count estimated by qPCR for three groups of lactate-utilizing *Lachnospiraceae*, total bacteria, and methanogenic archaea in fermentors inoculated with (a) D7 and (b) D19 microbiota at pH 6.5, pH 5.5, and a repeat run at pH 5.5, each in the presence of 0, 10, and 20 mM lactate. Download FIG S4, PDF file, 0.1 MB. Copyright © 2020 Wang et al. 2020 Wang et al. This content is distributed under the terms of the Creative Commons Attribution 4.0 International license.

Sequence read counts corresponding to these LUB were broadly in accordance with the data obtained by qPCR (see Table S2). Proportional abundances of OTUs 0063 and 0073, both corresponding to *A. hallii*, and OTU0034 (A. hadrus) increased on average concomitantly with lactate concentrations at pH 6.5. Although LEfSE *P values* for all of these OTUs were low (*P* = 0.007, 0.005, and 0.001, respectively), none were deemed significant after correction for the FDR using the Benjamini-Hochberg method (Table S2a). Other potential lactate-utilizers were not detected in all three fecal microbiota donor samples, but included OTU0157, corresponding to Coprococcus catus, that was proportionally enriched with lactate infusion in the D2 and D7 experiments, and *Veillonella* spp. that increased in the D7 experiment between 2 and 4 days at pH 6.5 (Table S2a). The behavior of LUB at pH 5.5 (Table S2b) was complicated by the effects of lactate growth inhibition, as discussed below, although increases were seen with 10 mM lactate infusion. Furthermore, LUB populations monitored by qPCR (Fig. S4) showed some variation between individual donor repeat experiments, which were performed several weeks apart, perhaps reflecting fluctuations in the initial species composition of the fecal inocula over time.

### Selective growth inhibition by lactate.

**FIG 6:**
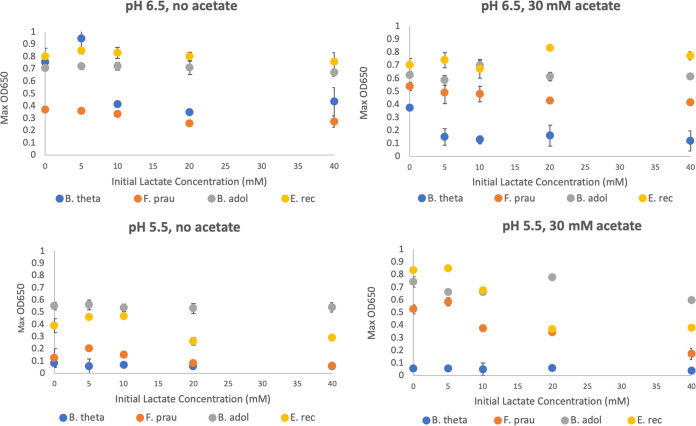
Growth (OD_650_) of four bacterial strains representing mathematical modeling microbial functional groups (MFGs) 1, 4, 5, and 6 in pure culture in YCFAG (10 mM glucose) medium containing different concentrations of lactate, with or without 30 mM acetate. B. theta, Bacteroides thetaiotaomicron; F. prau, Faecalibacterium prausnitzii; B. ado, Bifidobacterium adolescentis; E. rec, Eubacterium rectale.

The growth of representative strains of Bifidobacterium adolescentis, Eubacterium rectale, Faecalibacterium prausnitzii, and Bacteroides thetaiotaomicron was examined in pure culture with 10 mM glucose in the presence of 5 to 40 mM dl-lactate, either in the presence or absence of 30 mM acetate (Fig. 6). These experiments demonstrated different growth responses of the four species to lactate and acetate, and the results may help to explain the microbial community shifts observed in the fermentor experiments shown in Fig. 2 and 3. Growth of B. thetaiotaomicron at pH 6.5 was inhibited by concentrations of lactate of 10 mM or more, and growth inhibition was increased by the presence of 30 mM acetate. In contrast, growth of *B. adolescentis* was unaffected by lactate or acetate at either pH. Growth of E. rectale and *F. prausnitzii* in the presence of 30 mM acetate (required for optimal growth of these species) showed little effect of lactate up to 40 mM at pH 6.5 but was progressively decreased at pH 5.5.

### Theoretical modeling of the human colonic microbial community.

In order to gain better understanding of the factors underlying the shifts in microbiota composition and metabolites, we decided to apply the theoretical modeling approach developed by Kettle et al. (33) with slight modifications (see Materials and Methods) in parameter values. This model has now been made publicly available in the language R under the name microPop (34). The model, as applied to the human colonic microbiota, is based on the assumption of 10 microbial functional groups (MFGs; M1 to M10) whose substrate preferences, metabolic outputs, and responses to pH are all numerically defined. For further details about the model, see “Mathematical model” in Materials and Methods. Group M1 was taken to include all *Bacteroidetes* sequences while M4 (lactate producers) included all bifidobacteria and lactobacilli. *Firmicutes* were then split between four non-butyrate-producing groups (M2, M3, M7, and M9) and three butyrate-producing groups (M5, M6, and M8), as detailed in Table 1. Of these, only M7 (*Negativicutes* and Coprococcus catus) and M8 (*A. hallii* and *Anaerostipes* spp.) are assumed to be able to utilize lactate. The proportion of group M10 (methanogens) was based on estimates from qPCR (see Table S3a).

**TABLE 1:** Microbial functional groups used for model simulations^*a*^

MFG	Description	Taxa included
M1	Propionate producers	*Bacteroidetes*
M2	Starch degrading acetate producers	*Ruminococcus bromii*
M3	Acetate producers (nonacetogenic)	*Lachnospiraceae* plus *Ruminococcaceae* (minus M2, M5, M6, M8, and M9)
M4	Lactate producers	*Actinobacteria*, *Lactobacillaceae*, *Enterococcaceae*, *Streptococcaceae*, and *Peptococcaceae*
M5	Butyrate producers 1	*Roseburia* spp. (plus *Eubacterium rectale*)
M6	Butyrate producers 2	*Faecalibacterium* plus *Subdoligranulum*
M7	Lactate utilizers producing propionate	*Negativicutes* plus *Coprococcus catus*
M8	Lactate utilizers producing butyrate	*Anaerostipes* spp. plus *Anaerobutyricum* spp.
M9	Acetogens	*Blautia* spp.
M10	Methanogens	Methanogenic archaea

a MFG, microbial functional group.

10.1128/mSystems.00645-20.10 TABLE S3 Parameters used for mathematical modeling. (a) Assignment of 16S rRNA gene sequences to Microbial Functional Groups for fecal inocula from the three volunteers (D2, D7, and D19). Taxa (at the phylum family, genus, or species level) were assigned to groups M1 to M10 based on characteristics reported for cultured representatives. “%” refers to the percentage of total sequences for that sample (8.3 to 17.7% were unassigned). (b) Assumed maximal growth rate values (day^−1^) for MFGs used for model simulations. Assumptions (necessarily approximate) are informed as far as possible by experimental evidence from representative cultured isolates. (c) Assumed pH “corners” defining pH responses for MFGs. These define the effect of pH on growth rates. For pH values less than corner c1, or exceeding corner c4, the growth rates shown in Table S3b are assumed to be zero for the relevant MFG. For pH values between corners c2 and c3 growth rates are as shown in Table S3b. For pH values between c1 and c2 or between c3 and c4 growth rates are assumed to change linearly between those shown in Table S3b and zero. (d) Metabolic stoichiometries (relative number of moles of resource that are consumed or produced by each MFG). (e) Lactate concentration (mM) at which growth of each MFG is assumed to be suppressed by 50%. Download Table S3, DOCX file, 0.02 MB. Copyright © 2020 Wang et al. 2020 Wang et al. This content is distributed under the terms of the Creative Commons Attribution 4.0 International license.

**FIG 7:**
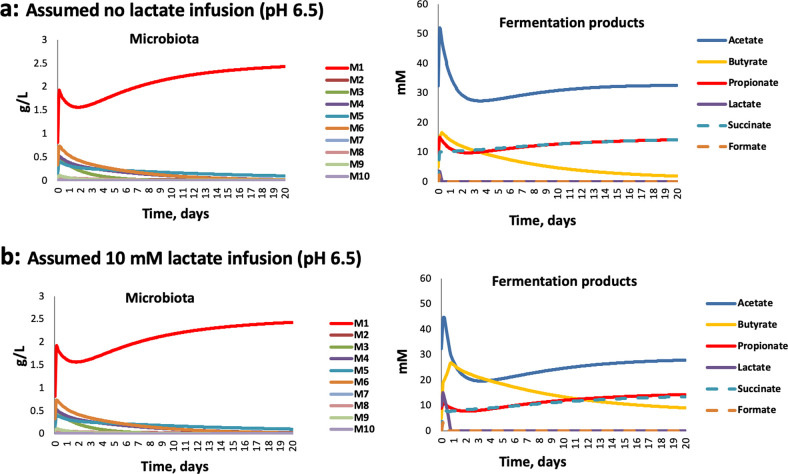
Simulating changes in microbiota composition and metabolite concentrations in fermentor experiments with D2 inocula at pH 6.5. (a) Assuming no lactate infusion; (b) assuming 10 mM continual lactate infusion.

**FIG 8:**
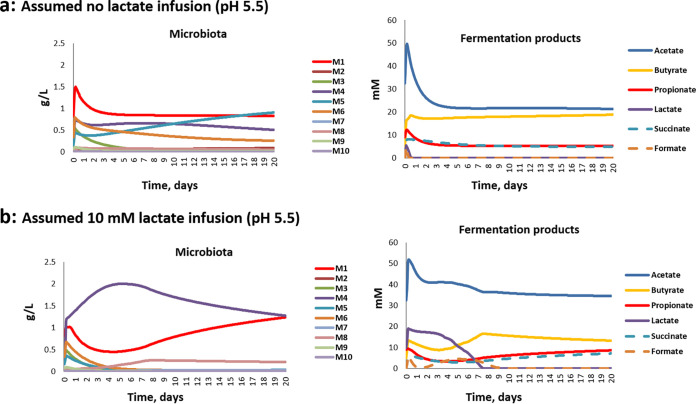
Simulating changes in microbiota composition and metabolite concentrations in fermentor experiments with D2 inocula at pH 5.5. (a) Assuming no lactate infusion; (b) assuming 10 mM continual lactate infusion.

Assumed maximum growth rates and pH responses used in the modeling are shown in Tables S3b and c, and stoichiometries are shown in Table S3d. Based on the pure culture experiments shown in Fig. 6, the model was modified to include noncompetitive inhibition of M1 (*Bacteroidetes*) by lactate (assumed *K_i_* = 5 mM) both at pH 6.5 and 5.5, and noncompetitive inhibition by lactate of all other groups apart from M4 (lactate producers such as bifidobacteria, assumed *K_i_* = 15 mM) at pH 5.5, but not at pH 6.5 (see Table S3e). This modification provided a good simulation of the microbiota and metabolite changes observed experimentally with and without lactate infusion (Fig. 7 and 8). Since there is only limited information on the inhibition of LUB by lactate (35), we assumed the same *K_i_* for groups M7 and M8 as for most other groups of *Firmicutes*. An alternative assumption of no inhibition of these two groups by lactate did not affect their modeled populations greatly but did improve lactate utilization and caused predicted changes in other MFGs, notably M1, M4, and M5 (see Fig. S5a and b). Importantly, simulation was very poor if no lactate inhibition was assumed, further indicating the critical role of lactate on overall microbial community dynamics (Fig. S5c).

10.1128/mSystems.00645-20.5 FIG S5 Effect of building lactate inhibition into the model. Modeling is shown here for the D2 microbial community in continuous culture held at pH 5.5 with 10 mM lactate infusion: (a) with inhibition by lactate assumed for all groups except M4, as shown in Table S3e (1); (b) inhibition by lactate assumed for groups M1, M2, M3, M5, M6, M9, and M10 (as in Table S3e), but not for LUB (M7 and M8) (2); (c) no lactate growth inhibition assumed. Lactate inhibition is predicted to have a pronounced effect on outcomes. Download FIG S5, TIF file, 0.4 MB. Copyright © 2020 Wang et al. 2020 Wang et al. This content is distributed under the terms of the Creative Commons Attribution 4.0 International license.

Simulations at pH 6.5 predicted the dominance of *Bacteroides* (M1) that was observed both in these experiments and in previous experiments (2, 3, 29) (Fig. 7). As explained previously (33), the impact of lower pH is effectively modeled by assuming a lower tolerance of mildly acidic pH for *Bacteroidetes* (group M1) compared to *Firmicutes* (groups M2, M3, M5, M6, M7, M8, and M9) or lactic acid bacteria, including bifidobacteria (M4). These pH responses (Table S3c) are based broadly on the experimental data of Duncan et al. (30). For the D2 experiments, these assumptions led to a decrease in the dominance of M1 (*Bacteroidetes*) when the pH shifted to pH 5.5, as noted previously (2, 33) (Fig. 8). It should be mentioned that we did not build an initial growth lag after inoculation into our modeling, although this could plausibly occur after the introduction of non-actively growing cells from fecal inocula into the fermentor medium. Such a growth lag might explain why the initial surge of M1 in the model runs was not observed experimentally.

### Predicted impact of initial microbiota composition, including the presence of lactate-utilizing bacteria, on the stability of the microbial ecosystem.

**FIG 9:**
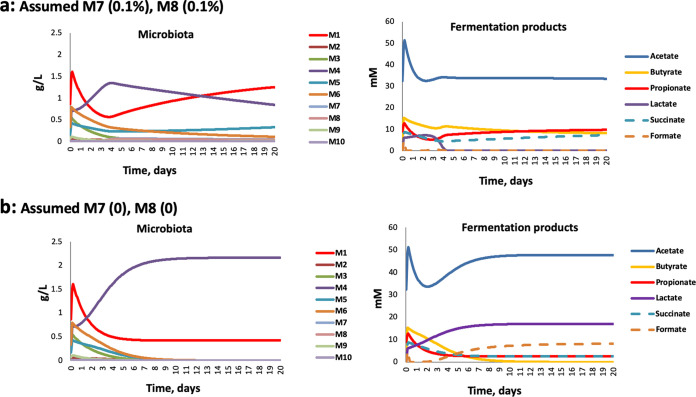
Simulation results showing effect of varying the proportional abundance of lactate-utilizers within the microbiota upon community stability and metabolite profiles at pH 5.5, assuming no additional lactate infusion, only production by the endogenous microbiota. (a) Assuming lactate utilizers at 0.2% of the total microbial community; (b) assuming lactate utilizers absent.

Variation in the experimental inocula between the three donors had little impact when modeled at pH 6.5 with no infusion of lactate (see Fig. S6), as was also observed in the actual fermentor experiments shown in Fig. 2a. The presence or absence of methanogens in the inoculum was also predicted to have relatively little impact, except on the levels of formate (Fig. S7). With lactate inhibition included in the model (see above), we found that progressively decreasing the proportion of the two groups of LUB (M7+M8) bacteria in the D2 community resulted in a failure to utilize lactate and caused a dramatic community shift, similar to that observed experimentally (Fig. 9). Decreasing the theoretical LUB population resulted in a switch toward a lactate-producing (M4)-dominated microbiota with a concomitant accumulation of lactate. This is likely to reflect the changing balance between the initial rates of lactate production and utilization, which together determine the lactate concentration and therefore the relative growth rates of lactate producers and lactate utilizers.

10.1128/mSystems.00645-20.6 FIG S6 Modeled outcomes for the three microbial communities at pH 6.5 (no lactate input). Modeling is based on the input % representation for the 10 MFGs given in Table S3a. It is evident that the inoculum variation was predicted to have little effect under these conditions. Download FIG S6, TIF file, 0.3 MB. Copyright © 2020 Wang et al. 2020 Wang et al. This content is distributed under the terms of the Creative Commons Attribution 4.0 International license.

10.1128/mSystems.00645-20.7 FIG S7 Modeling the predicted impact of M10 (methanogens) population on microbiota profiles and metabolites for fermentors inoculated with D2 microbiota. Results shown are the predictions for fermentors with no lactate infusion, at pH 6.5. (a) Modeling with no methanogens present; (b) modeling with methanogens present as 0.6% of the total microbial cell count. Download FIG S7, TIF file, 0.3 MB. Copyright © 2020 Wang et al. 2020 Wang et al. This content is distributed under the terms of the Creative Commons Attribution 4.0 International license.

In summary, our interpretation of the experimental data is as follows. For D2 inocula, we hypothesize that the initial lactate-utilizing activity was sufficient to prevent an excessive early increase in lactate within the fermentor at pH 5.5, and the system shows a balance of metabolite formation somewhat similar to that seen at the higher pH (6.5). The lower LUB activity within the D7 and D19 inocula may be part of the explanation for the pH dependent ability of these communities to utilize lactate in the absence of lactate infusion, as discussed below. Interestingly, the reversal of microbiota perturbations at some points by infusion of 10 mM lactate was also predicted by the model. This may be explained by stimulation of certain LUB populations by the presence of noninhibitory concentrations of lactate.

## DISCUSSION

The deleterious phenomenon of lactic acidosis has long been recognized in the rumen and in monogastric farm animals (36). Acidosis is generally triggered by dietary change, resulting in the replacement of a microbial community dominated by obligate anaerobic bacteria with one dominated by lactic acid bacteria (37). In ruminants it is considered that the promotion of lactate-producing bacteria such as Streptococcus bovis by high-starch diets results in a rise in lactate concentrations that decreases rumen pH as a result of the low pKa of lactic acid. This rapidly inhibits the growth of many obligate anaerobes, including lactic acid-utilizing bacteria (LUB) and creates conditions that further favor lactic acid producers. Similar events can occur in the human intestine, as for example in the extreme case of short bowel syndrome (38, 39).

We found previously in *in vitro* batch culture incubations with human fecal inocula, that an initially low pH (5.2) abolished production of butyrate and propionate, with lactate and acetate becoming the major products, whereas production of propionate and butyrate occurred with little or no lactate accumulation when the initial pH was set at pH 5.8 or 6.5 (24). Here, in continuous culture, pH 5.5 resulted in suppression of butyrate and propionate formation for two of the three fecal microbiota donors examined. However, the present investigations reveal that acidic pH is not the only factor involved in lactate-induced microbial community perturbation. Our results indicate that lactate can exert a selective antimicrobial effect at concentrations as low as 5 mM. Lactic acid bacteria are themselves relatively insensitive to lactate, as might be anticipated (40). Previous work has also demonstrated that certain *Proteobacteria* species, including some pathogens such as *Salmonella* Typhimurium, are able to effectively utilize lactate for growth (15). On the other hand, growth of representatives of the normally dominant *Bacteroidetes* and *Firmicutes* phyla was found here to be highly sensitive to lactate. The growth of B. thetaiotaomicron, for example, was inhibited in pure culture at pH 6.5 by 5 mM lactate in the presence of 30 mM acetate (Fig. 6). In contrast, *Bacteroides* dominated the fermentor communities supplied with lactate at pH 6.5 (Fig. 2), but this was in the absence of lactate accumulation, which we assume was prevented by the activity of LUB. The consequences of lactate inhibition for the fermentor community became evident at pH 5.5, however, when more groups were affected and LUB activity may be compromised. Lactate inhibition therefore explains why the accumulation of lactate can lead to a rapid shift in overall microbiota community composition from a community that was dominated by *Bacteroides* and *Firmicutes* to one dominated by lactic acid bacteria and *Proteobacteria*, as observed here. This interpretation is also strongly supported by the theoretical modeling performed here, which was found to reproduce the behavior of the observed fermentor microbial communities only when lactate inhibition was assumed.

It would seem obvious that infusion of lactate should further destabilize the microbiota, and this was indeed observed initially. On the other hand, 10 mM lactate infusion led to subsequent recovery of butyrate and/or propionate production by day 4 at pH 5.5. This apparent conundrum can, however, be understood if we consider the populations of LUB within the community. A crucial role for LUB in community stability is predicted by the theoretical modeling performed here (Fig. 9). First, we could show that a relatively small population of LUB appears to be sufficient to convert lactate infused at up to 20 mM into SCFAs and thereby prevent acidosis, provided that the pH remains close to neutrality. Much lower populations of LUB however resulted in a failure of lactate-utilization at pH 5.5, even with no infusion of lactate. Similarly, in our fermentor experiments, LUB populations did not decline when the pH was held at 6.5, but some dramatic decreases were seen with the pH at 5.5. This behavior was complicated by donor variation and the possible promotion of some groups by lactate infusion. We note, however, that the D2 experiments showed no decrease in LUB populations at pH 5.5 (with no lactate infusion) and no accumulation of lactate. Whereas the D7 and D19 experiments showed decreased LUB and lactate accumulation at pH 5.5, even with no lactate infusion. Interindividual variation in these functional groups of bacteria within the colonic microbiota is therefore likely to be a critical factor determining the stability of the whole community and its metabolic outputs. The growth responses of different LUB species to pH and to lactate will clearly merit more detailed experimental investigation in the future.

Remarkably, these communities often showed the ability to recover from the perturbed microbiota state when lactate infusion was maintained. This behavior was seen with all three fecal microbiota studied when supplied with 10 mM lactate and can probably be explained by increased lactate utilization resulting from selection for LUB. In the D7 experiments, recovery coincided with increased proportional abundance of *Veillonella* within the community after 2 days. It is less clear which LUB were involved in the case of D2, although *A. hadrus* and *C. catus* were proportionally increased at 48 h. It is important to stress, however, that we are not currently able to identify all LUB only from 16S rRNA gene sequences or genome sequences. There are likely to be unidentified LUB within the community, while conversely some strains belonging to taxa known to contain LUB may lack the activity. The identification of LUB activity must therefore be considered inexact at present. Information is particularly scarce for *Negativicutes* from the human colon, although there are many constituent taxa, such as *Megasphaera*, *Phascolarctobacterium* and *Veillonella*, that have previously been shown to be able to utilize lactate in order to produce propionate (22).

In addition, many *Proteobacteria*, including potential pathogens such as *Salmonella* (15) and sulfate-reducing bacteria (10), are known to utilize lactate. Lactate utilization by *Proteobacteria* is so far reported to involve oxygen, nitrate, or sulfate as terminal electron acceptors, although increases in *Proteobacteria* were observed in this study under anaerobic conditions. *Salmonella* infection is reported to alter host cell metabolism resulting in increased supply of lactate, nitrate, and oxygen from host tissues, allowing *Salmonella* Typhimurium to exploit lactate as an energy source for growth in the gut (15). At the same time, *Salmonella* infection is accompanied by decreased supply of butyrate, a regulator of host cell metabolism, from the colonic microbiota (41). While this suppression of butyrate-producing bacteria may be due in part to host immune responses (42), our present findings indicate that the impact of increased lactate influx upon the microbiota is also likely to be a key factor. Thus, increased lactate and decreased butyrate production are likely to combine to promote highly deleterious outcomes, including the promotion of pathogens and the production of toxic hydrogen sulfide (10, 16, 43). In the absence of pH control, the low pKa of lactate tends to decrease the colonic pH, which in turn tends to further promote microbial lactate production (24).

This analysis represents an important extension of our theoretical model of the human colonic microbiota previously described in Kettle et al. (33) using microPop (34). By incorporating selective inhibition by lactate into the model, we have been able to predict chaotic behavior in the system when challenged by an inhibitor supplied externally or generated endogenously. It is easy to see that the same equations can be readily applied in the future to considering the effects of other inhibitors such as bile acids and antibiotics as soon as the necessary information on selective inhibition of different microbial groups is available. The value of theoretical modeling for both predicting and explaining shifts in microbiota composition and metabolism has been illustrated here.

In conclusion, we have presented new experimental and theoretical evidence here that establishes the combined major impact of pH, lactate concentration, and the presence of LUB on the stability of human gut-derived microbial communities. Lactate appears to be an important “tipping element” (44) in the colonic ecosystem, particularly under conditions of lower pH. Increasing lactate concentration can promote a major switch in community composition and metabolite outputs, in which the sensitivity of different bacteria to decreasing pH and to lactate growth inhibition are both key factors. These findings are also highly relevant to the rumen, where lactate production and utilization has been shown to be a key factor not only in acidosis but also in differences between high and low productivity (and low- and high-methane-producing) animals (45, 46). Importantly, this work also highlights the potential for using LUB as novel therapeutic probiotics with the aim of restoring healthy microbiota composition and metabolism in perturbed states in humans. *Anaerobutyricum* (formerly *Eubacterium*) *hallii* has already been used experimentally as a therapeutic in a mouse model (47), but the potential of other (perhaps multiple) commensal LUB also appears to be well worth exploring. Improving our knowledge of the bacterial populations responsible for lactate utilization appears to be crucial to understanding interindividual differences in gut microbiota stability and responses to perturbations.

## MATERIALS AND METHODS

### Bacterial strains, growth medium, and conditions.

Four bacterial strains were tested for their ability to grow and utilize dl-lactate when added to growth medium with two initial pH values (pH 6.5 and pH 5.5). The four strains tested included Bacteroides thetaiotaomicron B5482 (DSM2079^T^) from Deutsche Sammlung von Mikroorganismen und Zellkulturen (DSMZ). The other strains (Faecalibacterium prausnitzii A2-165, Bifidobacterium adolescentis L2-32, and Eubacterium rectale A1-86) were all isolated at the Rowett Institute, University of Aberdeen. Cultures for growth studies were prepared by growing the inoculum on M2GSC medium (48) for 20 to 24 h at 37°C under CO_2_.

### Monococulture incubations.

Batch culture incubations were performed using anaerobic YCFA medium as described by Lopez-Siles et al. (49). The medium was adjusted to give two initial start pH values of 6.5 ± 0.1 and 5.5 ± 0.1 and dispensed in 7.5 ml volumes into Hungate tubes under a stream of CO_2_. The medium was heat sterilized at 121°C (15 min) prior to inoculation. The medium contained glucose (10 mM), either with or without 30 mM acetate and variable levels of lactate (0, 5, 10, 20, and 40 mM). After the medium was cooled, heat-labile vitamins were added. The tubes of medium were inoculated with 75 μl of overnight cultures pregrown in M2GSC medium. After inoculation, triplicate replicates of the tubes were incubated up to 48 h at 37°C in a water bath. Growth was determined using a spectrophotometer to monitor the optical density at 650 nm (OD_650_).

### Human colonic microbiota in continuous culture.

Single-stage fermentor systems were prepared largely as described previously (29) with a growth medium based on that of Macfarlane et al. (50). The carbon sources present in the mixed substrate medium were potato starch (0.5%) with lesser amounts of xylan, pectin, amylopectin, and arabinogalactan (each provided at 0.06% final concentration) and 0.2% peptides. To support the growth of species with a metabolic requirement for acetate (e.g., many LUB, as well as *Roseburia* and *Faecalibacterium* species), this SCFA was also added to the feed vessels at a concentration of 30 mM. For each individual set of fermentor runs, three parallel fermentors were provided with a final concentration of either 10 mM (“medium”) or 20 mM (“high”) dl-lactate in the feed vessels (or no added lactate as a control [“low”]). The volume of the fermentor culture vessel was kept constant at 250 ml, with an exchange of fresh growth medium of the same volume, which equates to one turnover per day, giving a dilution rate of 0.042 h^−1^. All fermentors were maintained at a constant temperature of 37°C throughout the experiments using a thermal jacket. Separate experiments were also conducted at which the pH was kept constant at either pH 5.5 ± 0.2 or pH 6.5 ± 0.2.

The fermentor vessels were inoculated with mixed human fecal microbiota, which was prepared by mixing fresh feces in 50 mM phosphate buffer containing 0.05% cysteine (pH 6.5) under CO_2_ to give a final concentration of 20% (wet weight/vol). The inoculum was added to the fermentor vessels to give a final concentration of 2% (wt/vol) feces. The experiments were replicated with fecal samples provided by three different healthy donors (donor 2, donor 7, and donor 19). Two of the donors were female (donors 2 and 19; aged 54 and 53 years old, respectively), and donor 7 was male, aged 64 years old. The donors consumed a habitual western style diet and had not taken antibiotics in the 3 months prior to providing the samples. Ethical approval for the sample collection was provided by the Rowett Institute’s internal ethical review panel (study number 5946). Samples were collected from the initial fecal inocula (ino) and from the fermentor vessels just after inoculation (t0/0h), 8 h after inoculation (8h), 24 h after inoculation (1d), and then daily thereafter at 24-h intervals through day 4 (2d, 3d, and 4d). It should be noted that the t0/0h measurements for acetate and lactate were typically at slightly lower concentrations than included in the feed vessels due to initial dilution of fermentor contents as a result of addition of fecal slurry and/or of buffer solutions to stabilize starting pH. Thereafter, and for the duration of the experiments, vessels were continuously infused with 30 mM acetate and the various concentrations of lactate as described above.

### DNA extractions and PCR amplification from fermentor samples.

Aliquots (460 μl) of samples collected from the model fermentor system were centrifuged at 10,000 × *g* for 10 min to pellet bacterial cells, and the pellet was then resuspended in 460 μl of PBS-glycerol buffer (containing 20% glycerol). Samples were stored at –20°C until DNA was extracted, at which time samples were thawed on ice, and the contents transferred to a FastDNA Spin kit lysing matrix E tube. Sodium phosphate buffer and MT buffer were added to the tubes, and DNA was extracted according to manufacturer’s instructions (MP Biomedicals, Illkirch, France). DNA was eluted in 50 μl of FastPrep elution buffer.

### PCR amplification of 16S rRNA genes and Illumina MiSeq sequencing.

The extracted DNA was used as a template for PCR amplification of the V1-V2 region of bacterial 16S rRNA genes using the barcoded fusion primers MiSeq-27F (5′-AATGATACGGCGACCACCGAGATCTACACTATGGTAATTCCAGMGTTYGATYMTGGCTCAG-3′) and MiSeq-338R (5′-CAAGCAGAAGACGGCATACGAGAT-barcode-AGTCAGTCAGAAGCTGCCTCCCGTAGGAGT-3′), which also contain adaptors for downstream Illumina MiSeq sequencing, as described previously (3). Each of the samples was amplified with a unique (12-base) barcoded reverse primer.

PCR amplification was undertaken with Q5 High-fidelity DNA polymerase (New England BioLabs). Each reaction mix contained DNA template (1 μl), 5× Q5 buffer (5 μl), 10 mM deoxynucleoside triphosphates (0.5 μl), 10 μM F primer (1.25 μl), 10 μM R primer (1.25 μl), Q5 *Taq* (0.25 μl), and molecular-grade nuclease-free water (15.75 μl) to a final volume of 25 μl. The PCR amplification conditions were as follows: 2 min at 98°C; 20 cycles of 30 s at 98°C, 30 s at 50°C, and 90 s at 72°C; ending with 5 min at 72°C and then a holding temperature at 10°C. Four PCRs were prepared for each DNA sample, and the quadruplicate reactions were pooled, ethanol precipitated, and quantified using a Qubit 2.0 fluorometer with a Qubit HS assay kit (Life Technologies, Ltd.). A sequencing master-mix was created using equimolar concentrations of DNA from each sample. Sequencing was carried out on Illumina MiSeq machines, using a 2 × 250-bp read length, at the Wellcome Sanger Institute (Cambridgeshire, UK) and the Centre for Genome Enabled Biology and Medicine (CGEBM; Aberdeen, UK).

### Analysis of 16S rRNA gene sequencing data.

The sequences obtained were analyzed using the mothur software package (51), with the forward and reverse reads first assembled into paired read contigs. Any paired contigs that were shorter than 270 bp, longer than 480 bp, contained ambiguous bases or contained homopolymeric stretches of longer than 7 bases were then removed. Unique sequences were grouped together and aligned against the SILVA reference database provided at the mothur website (https://mothur.org/wiki/Silva_reference_files). In order to reduce the impact of sequencing errors, preclustering (diffs = 3) was performed, and all reads that occurred less than three times across the entire data set were also removed. Reads classified as either chloroplast, mitochondria, eukaryote, or unknown sequences were removed from the data set, and then OTUs were generated at a 97% similarity cutoff level. All samples were rarefied to 7,095 reads to ensure equal sequencing depth for all comparisons (the median Good’s coverage estimate at this sequencing depth was 98.6% [range, 96.6 to 99.8%]). The final OTU-level results are shown in Table S1 in the supplemental material. OTU richness and Shannon and Inverse-Simpson diversity indices were calculated for each sample using mothur. Boxplots were created using BoxPlotR (52). Overall community clustering patterns were calculated by creating a Bray-Curtis dendrogram in mothur, which was visualized using iTOL (53). Significance was tested using the Parsimony command in mothur (*n* = 160 samples included in this analysis). To further visualize microbiota compositional differences between different donors and pHs, principle-component analysis (PCA) was conducted using only OTUs with a minimum 0.01% relative abundance (335/3,489 OTUs) using the online web portal Calypso, version 8.84 (54). OTU data were normalized using total sum normalization (TSS) and then transformed using cumulative-sum scaling and log_2_ transformation. Cumulative-sum scaling (CSS) is a widely used method for normalizing microbial community compositional data. CSS corrects bias introduced by TSS (55). In Calypso, if CSS is selected, TSS normalized data are also log_2_ transformed to account for the nonnormal distribution of taxonomic counts data.

LEfSe (56) and Spearman correlation analyses to assess associations between different lactate infusion levels and microbiota composition at the two different pHs were carried out using MicrobiomeAnalyst (57). LEfSe generates both a *P* value and a Linear Discriminant Analysis (LDA) score, the latter as a guide to effect size. For completeness, we have reported both values for every taxa listed in Tables S2a and b in the supplemental material. A false discovery rate adjustment (Benjamini-Hochberg, using MicrobiomeAnalyst) was applied to the LEfSe *P* values, to reduce the risk of identifying false positives due to multiple comparisons. For the purposes of the LEfSe and Spearman correlation analyses, time was not considered as a factor, and the four different time points for each donor were instead classified into either “high,” “medium,” and “low” lactate groups.

### Quantitative real-time PCR analysis.

The DNA extractions for 16S rRNA gene sequencing were also used to generate qPCR results. qPCR was carried out as described previously (58), according to the following modifications: a total of 2 ng DNA, as determined using a Qubit 2.0 fluorometer (Life Technologies, Ltd.), was used per reaction for most samples. Low concentration samples were diluted 1:1 in herring sperm DNA, and 2 μl was added to the reaction. The final concentrations per reaction for the low concentration samples were as follows (all values are ng of DNA): D7_H_1d_5_5, 0.15; D7_H_2d_5_5, 0.34; D7_H_3d_5_5, 1.07; D7_H_4d_5_5, 2.44; D7_Rpt_M_5_5_0h, 0.71; D7_Rpt_M_5_5_2d, 0.98; D7_Rpt_H_5_5_0h, 1.98; D19_Rpt_L_5_5_2d, 2.08; and D19_Rpt_L_5_5_4d, 2.18). These variable input concentrations were factored into the final calculations of 16S rRNA gene copy numbers per sample volume.

### Short-chain fatty acid and lactate determinations.

The SCFA and lactate content of batch culture and fermentor samples were determined by capillary gas chromatography analysis after conversion to *t*-butylmethylsilyl derivatives (59). The lower limit for reliable detection of SCFA changes was 0.2 mM.

### Mathematical model.

The theoretical modeling approach developed previously by Kettle et al. (33) was applied, using the freeware R library microPop (34). The model, as applied to the human colonic microbiota in a continuous fermentor setting, is based on the assumption of 10 microbial functional groups (MFGs, M1 to M10) whose substrate preferences, metabolic outputs and responses to pH are all specified based on current microbiological knowledge. These relationships are captured in suitable mathematical formulations, which then form part of a system of ordinary differential equations (ODEs; full details in Kettle et al. [33]). Furthermore, within each MFG, 10 “strains” were generated whose characteristics were allowed to vary stochastically within a narrow range of the predefined group characteristics, resulting in a microbial community initially of 100 “strains.” For given fermentor conditions (such as flow rates, medium composition and pH control) and given initial microbial composition, the ODE system was then solved numerically using the R library microPop (34) to simulate how the microbial community evolves over time.

Initial bacterial community composition (see Table S3a in the supplemental material) was estimated from the 16S rRNA gene sequence data for the fecal inoculum samples (Table S1). Populations of methanogens (group M10) were estimated by qPCR rather than 16S rRNA gene sequence data (Table S3a), since these were not detected using the 16S rRNA gene PCR primers. Group M1 was taken to include all *Bacteroidetes* sequences, while M4 (lactate producers) included all bifidobacteria and lactobacilli and M10 methanogens. *Firmicutes* were then split between the four non-butyrate-producing groups M2, M3, M7, and M9 and the three butyrate-producing groups M5, M6 and M8, as detailed in Table 1. Of these, only M7 and M8 are assumed to be able to utilize lactate. The remaining sequences that could not be readily assigned to these groups (7 to 16%) were arbitrarily distributed between the nine bacterial MFGs in proportion to the abundance of each group. These included the *Proteobacteria*, which in view of their diversity and metabolic flexibility were not assigned to a single MFG. Populations of methanogens (group M10) were estimated by qPCR. For D7 and D19, methanogen populations in the inocula were very low and 0.1% of microbial biomass was assigned to the group M10. For D2, however, higher levels were detected, and the value of 0.6% was used for M10 in the inoculum.

Microbial parameters were modified slightly from those given in Kettle et al. (33). Assumed maximum growth rates and pH responses are shown in Table S3b and 3c, and stoichiometries in Table S3d. Two minor changes were made that simplify alternative pathway stoichiometries (Table S3d). First, a single stoichiometry is assumed for M5 (4 hexose plus 2 acetate give rise to 5 butyrate, 6 H_2_, 8 CO_2_ and 2 H_2_O). Second, only two of the three stoichiometries given by Kettle et al. (33) are assumed here for M9. Some adjustments were made to the maximum growth rates and pH responses assumed for the various MFGs in order to approximate the observed representation of the major MFGs (Table S3b and c). Half-saturation constants, which is the substrate concentration at which half the maximum growth rate is achieved, were set at 0.001 g/liter for all MFGs and all substrates. To generate a microbial population, within each MFG 10 strains were generated whose maximum growth rates (Table S3b) and half-saturation constants varied randomly within 10% of the predefined group values, and their pH curves (Table S3c) were allowed to shift randomly by up to 0.2 U.

For the simulated experimental parameters, vessel turnover was set to 1/d. The medium comprised protein at 2 g/liter, starch at 5.6 g/liter, nonstarch polysaccharides at 1.8 g/liter, and 32 mM acetate. Lactate concentration in the medium was set to either 0 or 10 mM. The amount of substrate in the vessel at time zero was assumed identical to that of the medium. It was assumed that the vessel was seeded with 2 g/liter of inoculum, with its composition based on 16S rRNA gene sequence data (see Table S3a).

The model was extended to allow for selective bacterial growth inhibition in the presence of lactate. This was achieved by multiplying the growth rate by a growth suppression factor, which was modeled as *K_i_*/(*K_i_* + L), where L is the lactate concentration (mM) in the vessel. *K_i_* is the lactate concentration at which growth is inhibited by 50% (see Table S3e) and was derived from experimental data (see above). All model results are presented as the average of 10 simulation runs. The biomass of each MFG was calculated by aggregating the estimated biomass of the individual strains in the corresponding MFG.

### Data availability.

Sequence data have been deposited in the European Nucleotide Archive and are available under study accession number PRJEB35259 and sample accession numbers ERS4125408 to ERS4125571 (see Table S1 in the supplemental material).
