# Supplementary figures and images for: Pivotal Roles for pH, Lactate, and Lactate-Utilizing Bacteria in the Stability of a Human Colonic Microbial Ecosystem
Source: mSystems. 2020 Sep 8;5(5):e00645-20. doi: 10.1128/mSystems.00645-20 (PMC7483512; doi:10.1128/mSystems.00645-20)

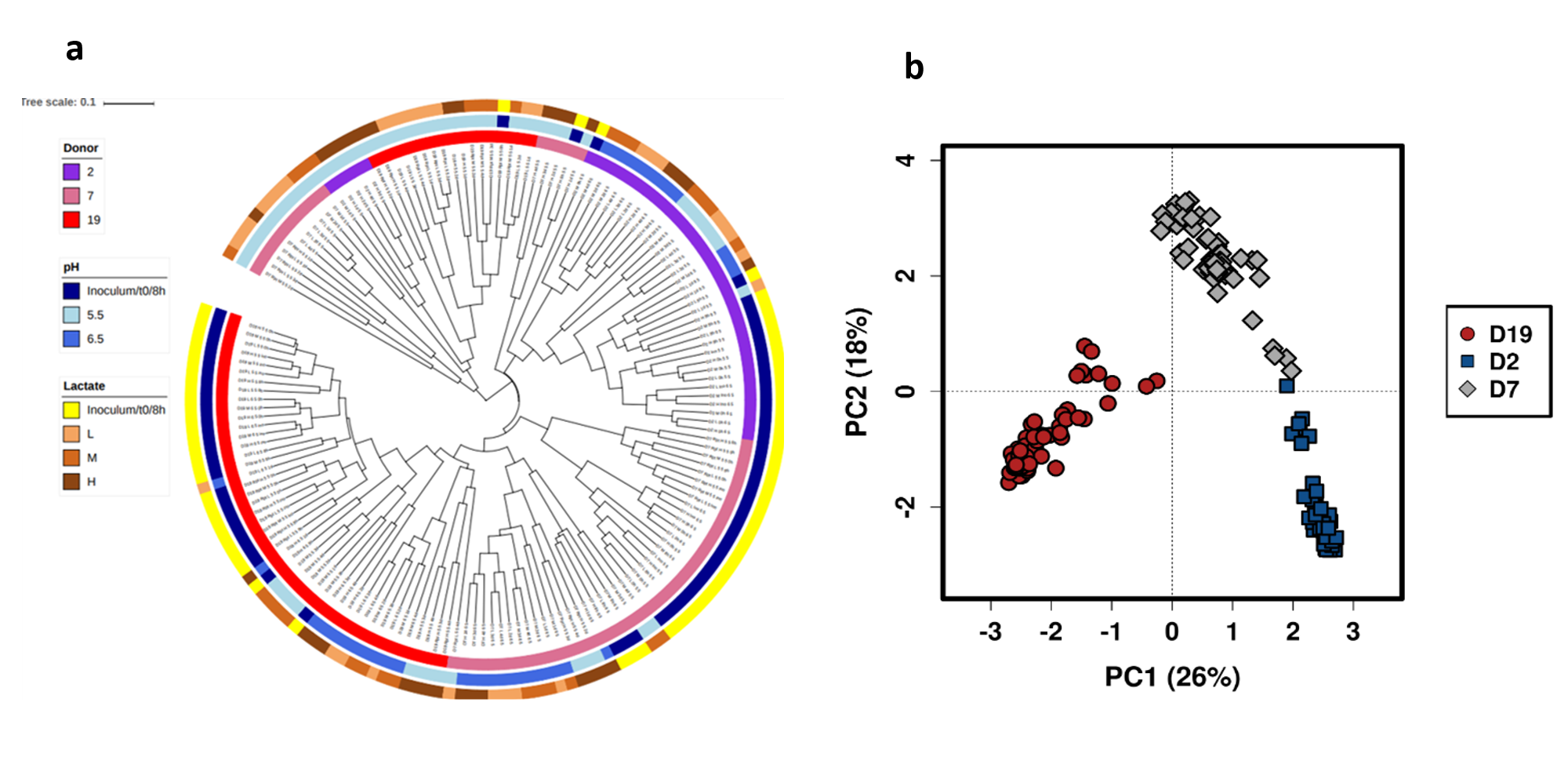

Supplement: FIG S1 [file mSystems.00645-20-sf001.tif]

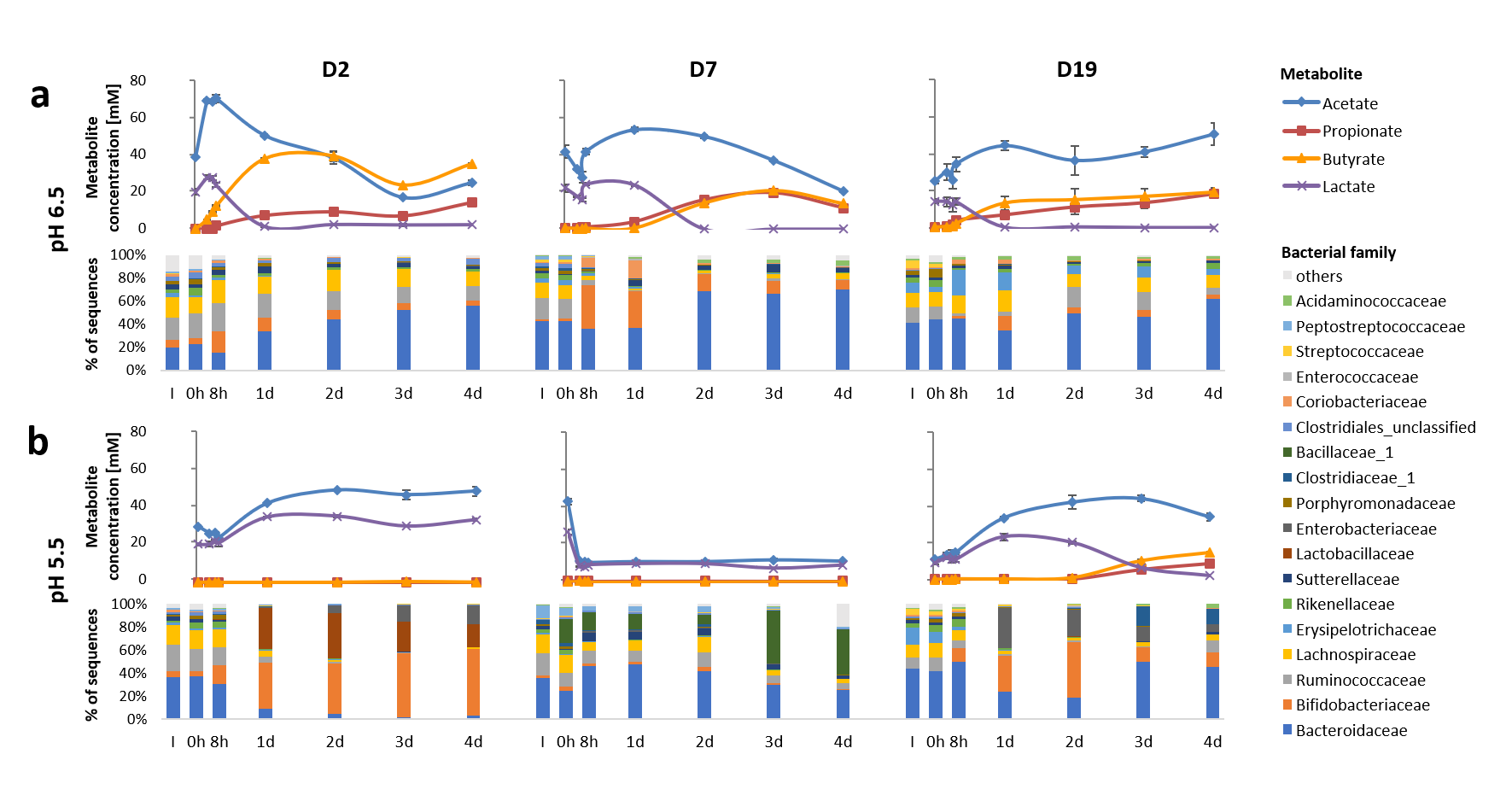

Supplement: FIG S2 [file mSystems.00645-20-sf002.tif]

**a**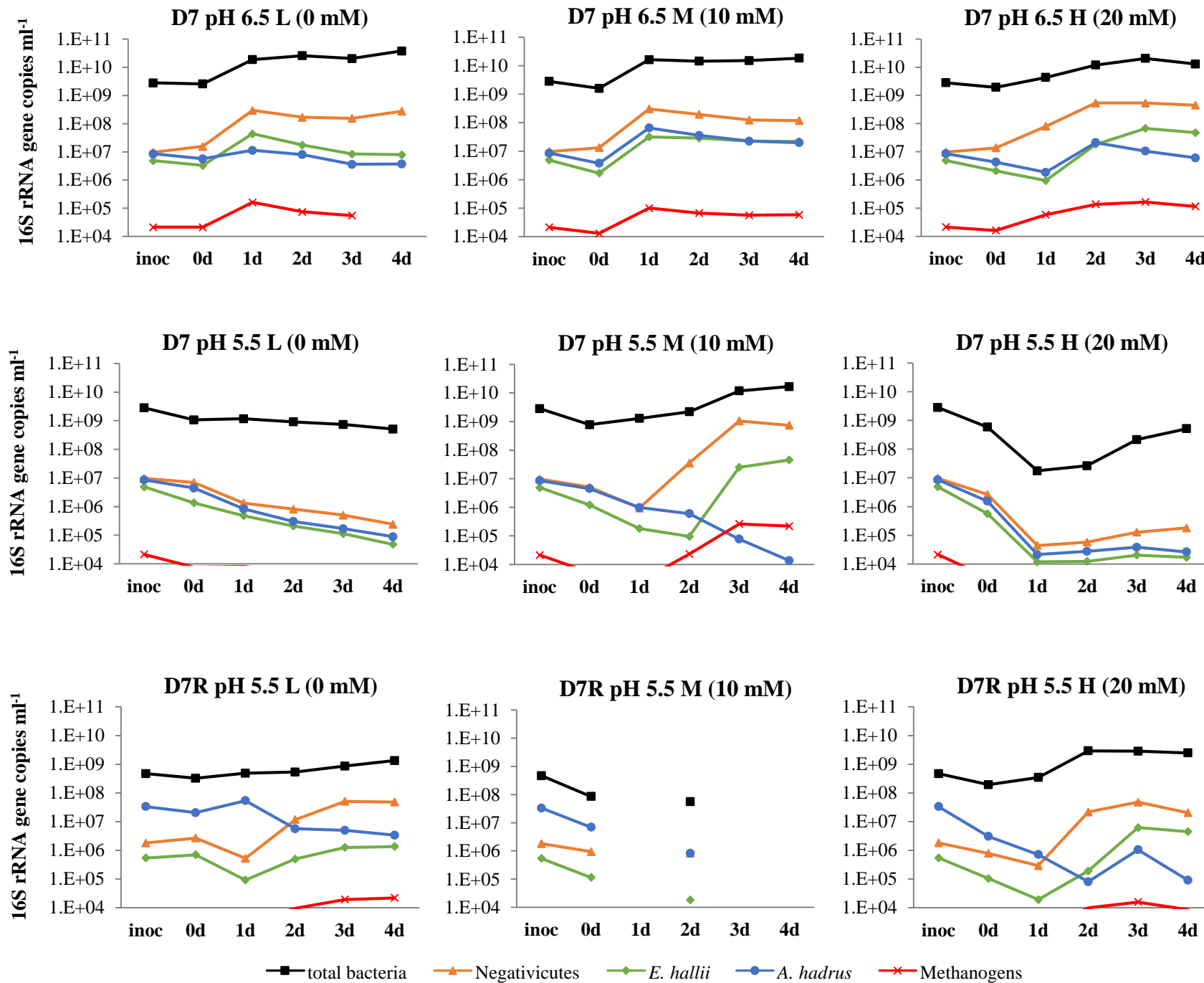

b

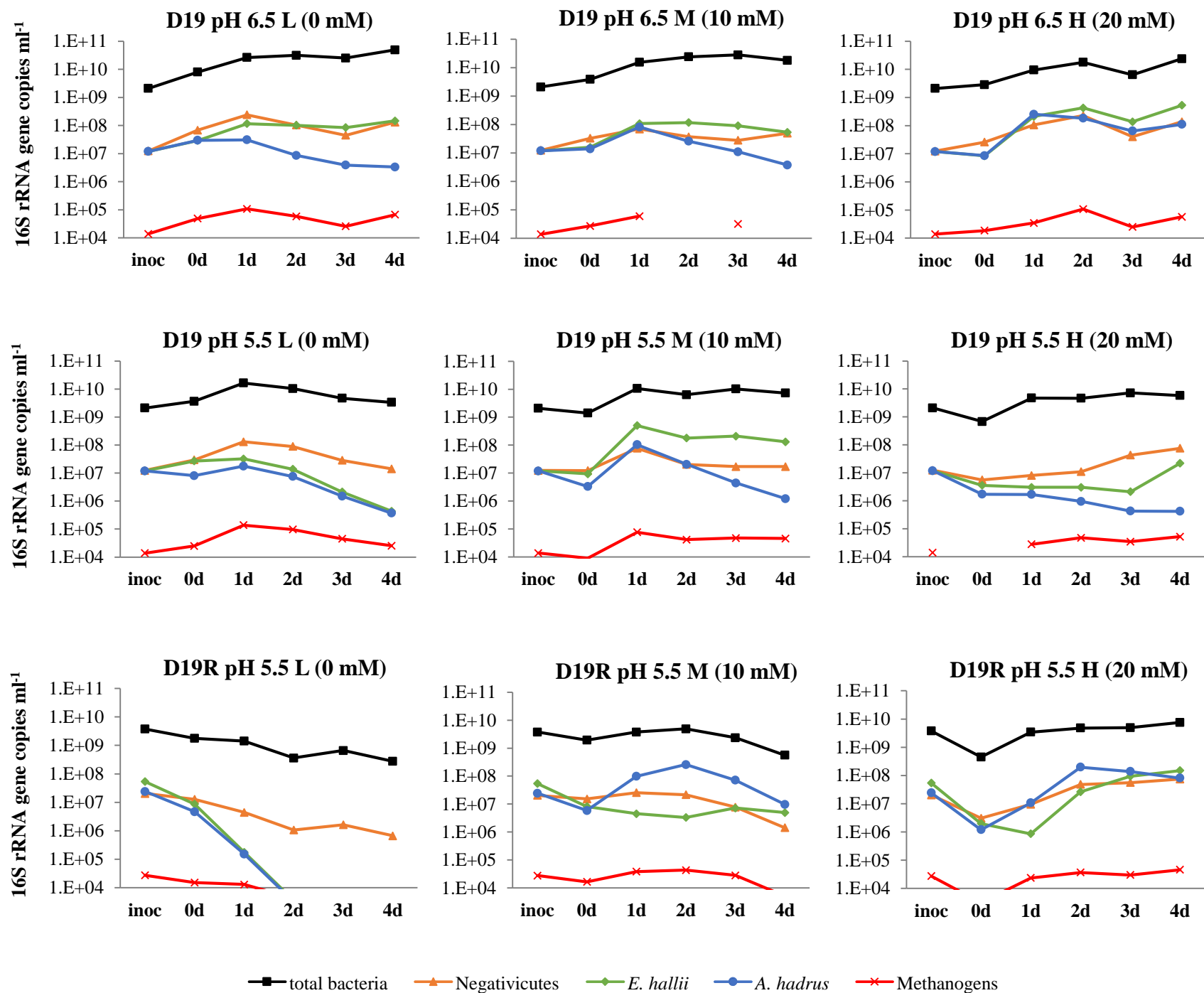

Supplement: FIG S4 [file mSystems.00645-20-sf004.pdf]

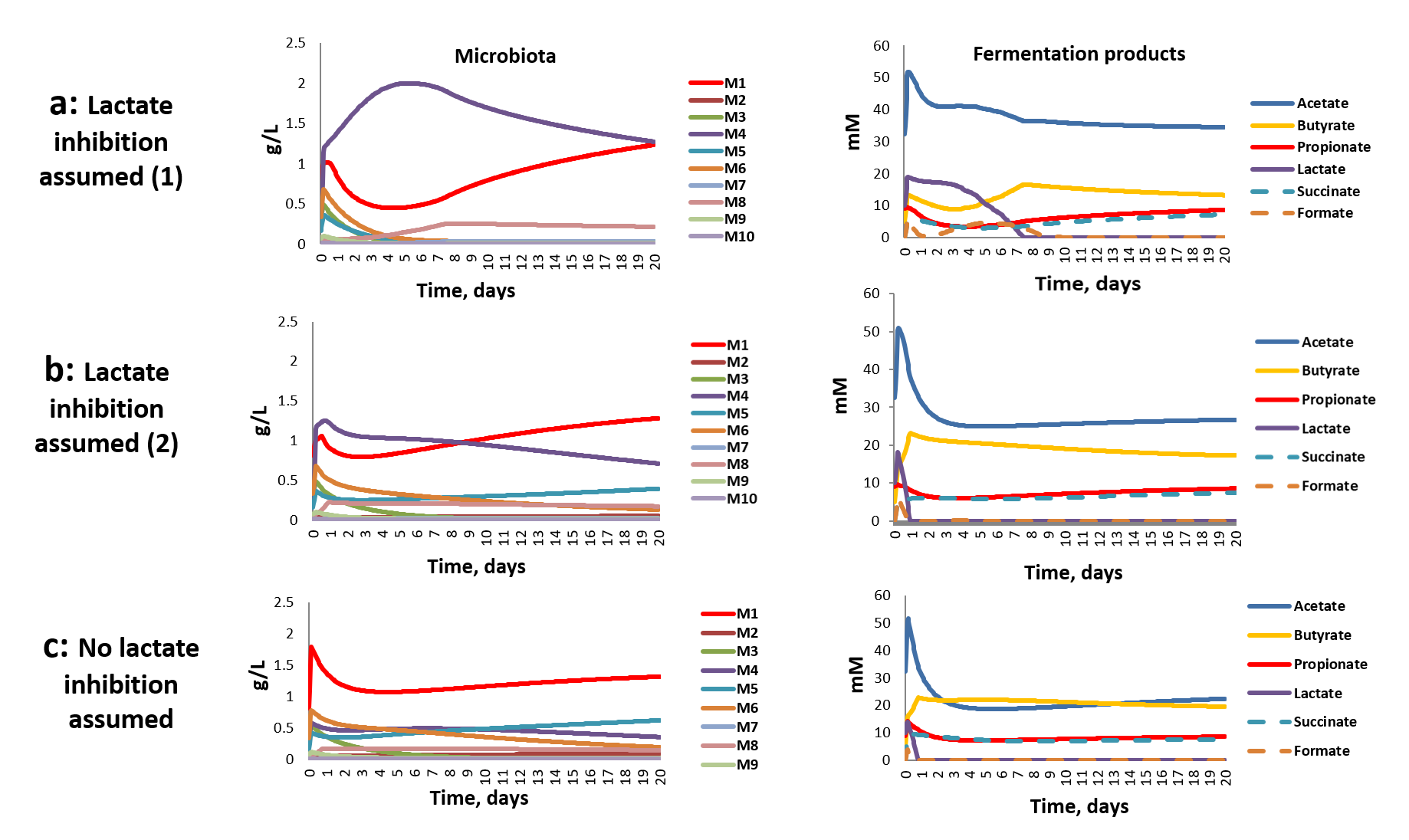

Supplement: FIG S5 [file mSystems.00645-20-sf005.tif]

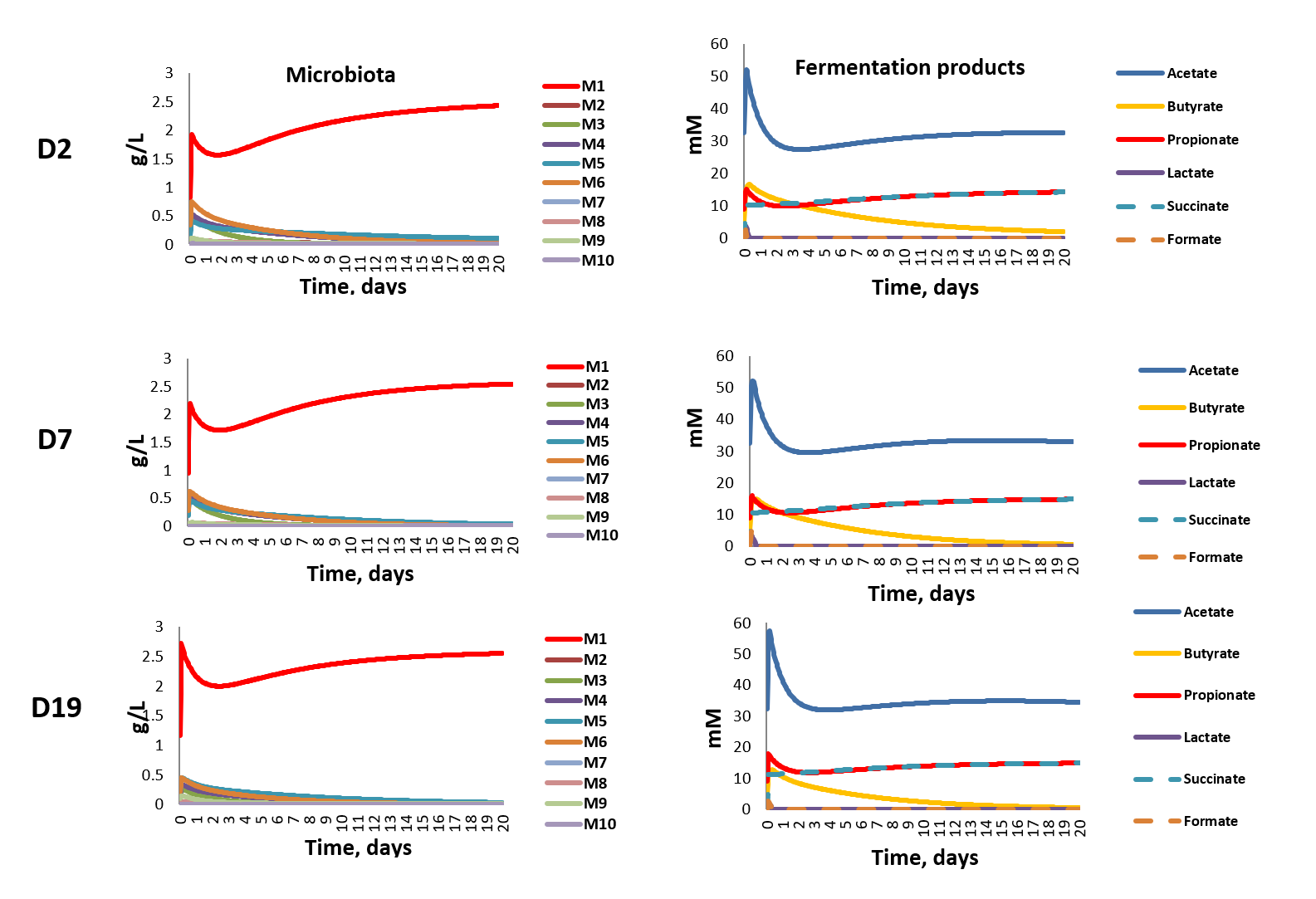

Supplement: FIG S6 [file mSystems.00645-20-sf006.tif]

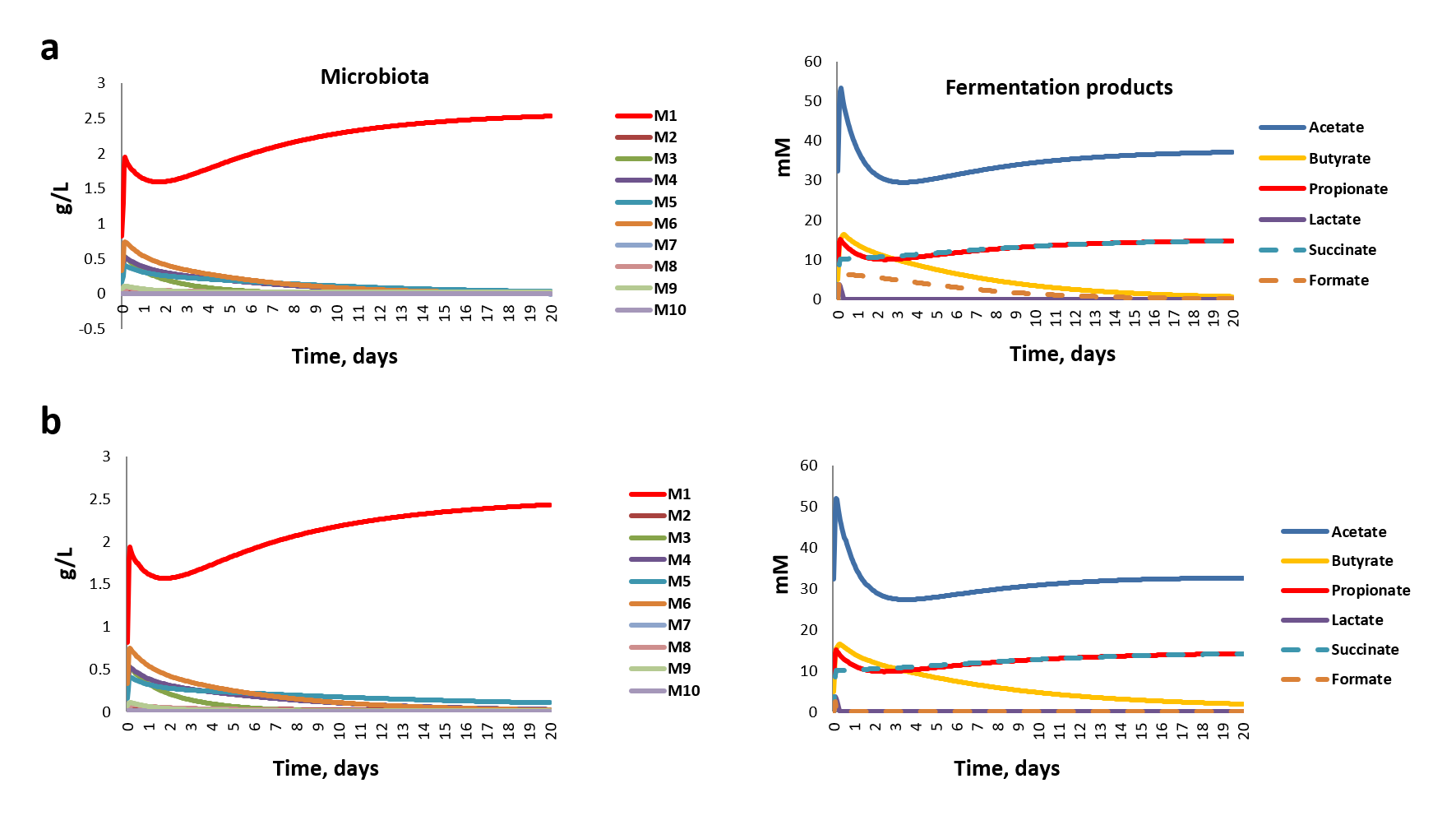

Supplement: FIG S7 [file mSystems.00645-20-sf007.tif]
