# Supplementary material for: Pivotal Roles for pH, Lactate, and Lactate-Utilizing Bacteria in the Stability of a Human Colonic Microbial Ecosystem
Source: mSystems. 2020 Sep 8;5(5):e00645-20. doi: 10.1128/mSystems.00645-20 (PMC7483512; doi:10.1128/mSystems.00645-20)
Supplement: FIG S3 [file mSystems.00645-20-sf003.pdf]

a

Run 1

D2

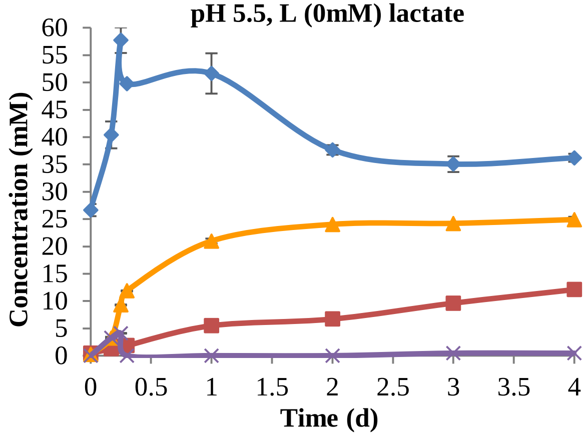

D2

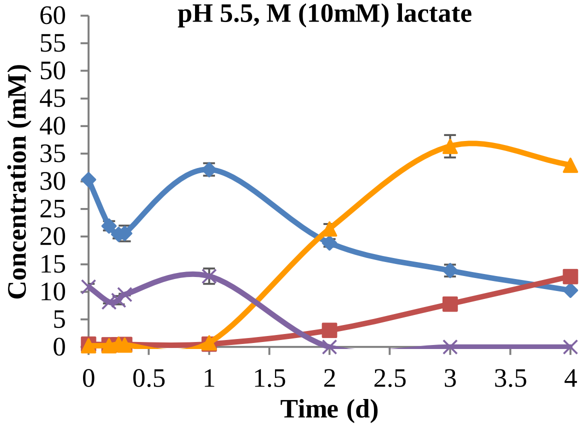

D2

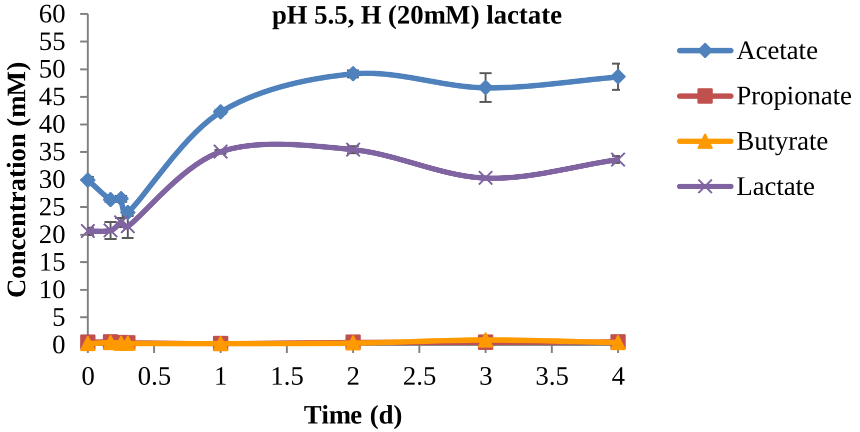

Run 2

(repeat 20mM Lactate)

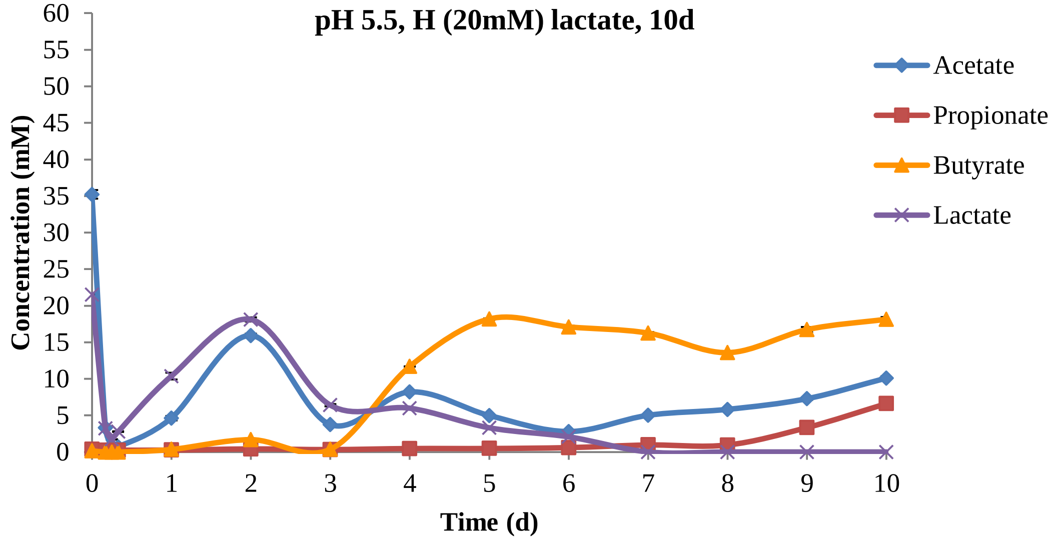

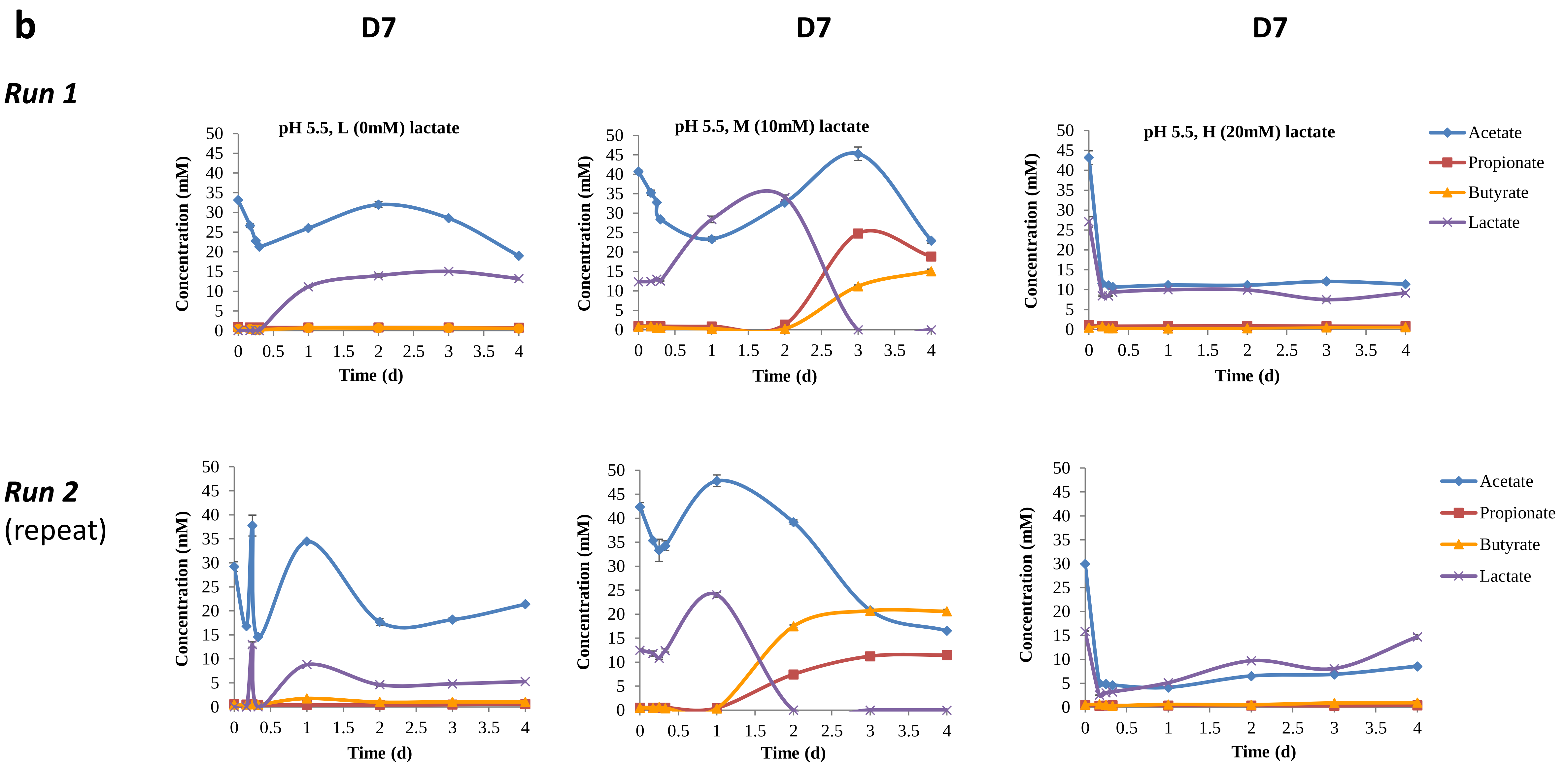
b
D7
D7
D7
Run 1
Run 2
(repeat)
pH 5.5, L (0mM) lactate
pH 5.5, M (10mM) lactate
pH 5.5, H (20mM) lactate
Concentration (mM)
Time (d)
Acetate
Propionate
Butyrate
Lactate

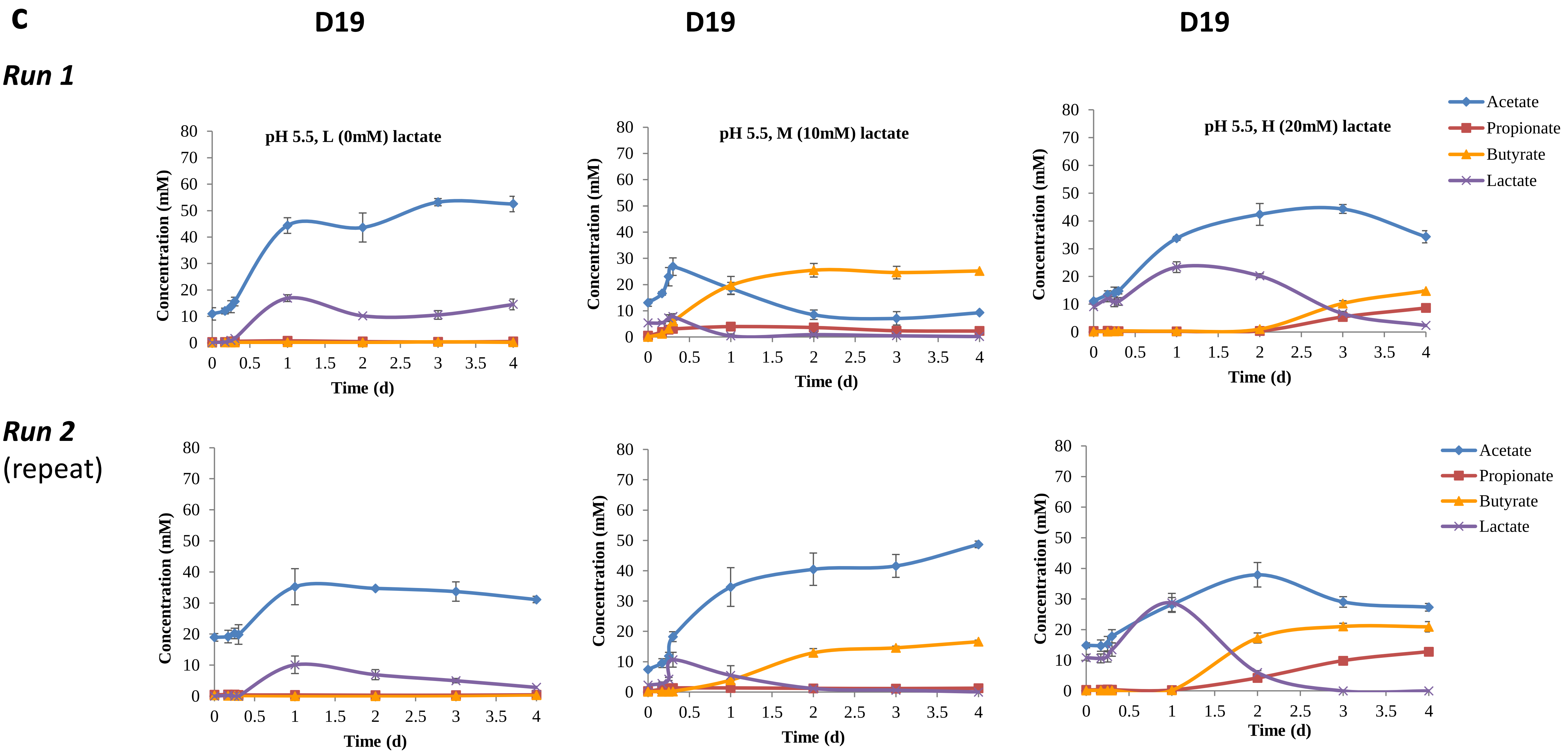
c
D19
D19
D19
Run 1
pH 5.5, L (0mM) lactate
pH 5.5, M (10mM) lactate
pH 5.5, H (20mM) lactate
Concentration (mM)
Time (d)
0
10
20
30
40
50
60
70
80
0
0.5
1
1.5
2
2.5
3
3.5
4
Acetate
Propionate
Butyrate
Lactate
Run 2
(repeat)
