# Supplementary material for: Pivotal Roles for pH, Lactate, and Lactate-Utilizing Bacteria in the Stability of a Human Colonic Microbial Ecosystem
Source: mSystems. 2020 Sep 8;5(5):e00645-20. doi: 10.1128/mSystems.00645-20 (PMC7483512; doi:10.1128/mSystems.00645-20)
Supplement: TABLE S3 [file mSystems.00645-20-st003.docx]

**Supplementary Table 3 (a to e): Modelling assumptions**

1. **Assignment of 16S rRNA gene sequences to Microbial Functional Groups**

Shown below for fecal inocula from the three volunteers (D2, D7, D19). Taxa (at the phylum family, genus or species level) were assigned to groups M1-10 based on characteristics reported for cultured representatives. “%” refers to % of total sequences for that sample (8.3% - 17.7% were unassigned).

|  | **Fecal inoculum (%)^1^** | | | **Approximate % (modelling)^2^** | | | | | |  |
| --- | --- | --- | --- | --- | --- | --- | --- | --- | --- | --- |
| **MFG** | **D2** | **D7** | **D19** | **D2** | **D7** | **D19** | | |  |  |
|  |  |  |  |  |  |  | | |  |  |
| M1 | 33.7 | 43.7 | 49.1 | 40.4 | 47.5 | 57.9 | | |  |  |
| M2 | 1.0 | <0.01 | <0.01 | 1.2 | 0 | 0 | | |  |  |
| M3 | 12.7 | 13.1 | 6.8 | 15.4 | 14.3 | 8.1 | | |  |  |
| M4 | 9.4 | 10.4 | 6.5 | 11.4 | 11.3 | 7.7 | | |  |  |
| M5 | 6.7 | 8.6 | 8.2 | 8.2 | 9.4 | 9.6 | | |  |  |
| M6 | 14.0 | 13.0 | 8.8 | 16.8 | 14.1 | 10.4 | | |  |  |
| M7 | 0.14 | 0.08 | 0.1 | 0.2 | 0.1 | 0.1 | | |  |  |
| M8 | 1.7 | 1.1 | 0.9 | 2.1 | 1.2 | 1.1 | | |  |  |
| M9 | 3.1 | 1.9 | 4.2 | 3.7 | 2 | 5 | | |  |  |
| M10 | 0^3^ | 0 | 0 | 0.6 | 0.1 | 0.1 | | |  |  |
| *Sum* | *82.4* | *91.9* | *84.6* | *100* | *100* | 100 | | |  |  |
|  |  |  |  |  |  |  |  |  |  |  |
|  |  |  |  |  |  |  |  |  |  |  |

1. Distribution of 16S rRNA gene sequences that could be assigned to MFGs.
2. % values used for modelling (after adjustment of bacterial 16S rRNA gene proportional values to 100% and inclusion of estimated methanogen populations)
3. Methanogen abundance estimated by qPCR (not sequencing, as they were not detected by the PCR primers used to generate 16S rRNA gene amplicons)
4. **Assumed maximal growth rate values (d^-1^) for MFGs used for model simulations.**

Assumptions (necessarily approximate) are informed as far as possible by experimental evidence from representative cultured isolates.

| MFG | Name | Protein | NSP^1^ | RS^1^ | Lactate | CO_2_ | Formate |
| --- | --- | --- | --- | --- | --- | --- | --- |
| M1 | Bacteroides | 9 | 16 | 16 |  |  |  |
| M2 | NoButyStarchDeg |  | 3 | 13 |  |  |  |
| M3 | NoButyFibreDeg |  | 16 | 6 |  |  |  |
| M4 | LactateProducers |  | 9 | 11 |  |  |  |
| M5 | ButyrateProducers1 |  | 9 | 12 |  |  |  |
| M6 | ButyrateProducers2 |  | 12 | 10 |  |  |  |
| M7 | PropionateProducers |  | 6 | 6 | 3 |  |  |
| M8 | ButyrateProducers3 |  | 6 | 6 | 3 |  |  |
| M9 | Acetogens |  | 6 | 6 |  | 2.4 | 2.4 |
| M10 | Methanogens |  |  |  |  | 2.4 | 2.4 |

^1^NSP = Non-starch polysaccharides. RS = Resistant starches.

1. **Assumed pH ‘corners’ defining pH responses for MFGs**

These define the effect of pH on growth rates. For pH values less than corner c1, or exceeding corner c4, the growth rates shown in Table S5 are assumed to be zero for the relevant MFG. For pH values between corners c2 and c3 growth rates are as shown in Table S5. For pH values between c1 and c2 or between c3 and c4 growth rates are assumed to change linearly between those shown in Table S5 and zero.

| MFG | Name | c1 | c2 | c3 | c4 |
| --- | --- | --- | --- | --- | --- |
| M1 | Bacteroides | 5.05 | 5.8 | 7.2 | 7.5 |
| M2 | NoButyStarchDeg | 4.75 | 5.5 | 7.2 | 7.5 |
| M3 | NoButyFibreDeg | 4.75 | 5.5 | 7.2 | 7.5 |
| M4 | LactateProducers | 4.5 | 5.25 | 7.2 | 7.5 |
| M5 | ButyrateProducers1 | 4.75 | 5.5 | 7.2 | 7.5 |
| M6 | ButyrateProducers2 | 4.75 | 5.5 | 7.2 | 7.5 |
| M7 | PropionateProducers | 4.75 | 5.5 | 7.2 | 7.5 |
| M8 | ButyrateProducers3 | 4.75 | 5.5 | 7.2 | 7.5 |
| M9 | Acetogens | 4.75 | 5.5 | 7.2 | 7.5 |
| M10 | Methanogens | 5.05 | 5.8 | 7.2 | 7.5 |

1. **Metabolic stoichiometries (relative number of moles of resource that are consumed or produced by each MFG)**

Note Hex is the sugar produced by the breakdown of carbohydrates, and all dietary carbohydrate substrates are regarded in terms of hexose equivalents. “Ac” = acetate, “Lac” = lactate, “For” = formate, “Prop” = propionate, “Bu” = butyrate, “Suc” = succinate, “EtOH” = ethanol.

|  |  | Resources | | | | | | | |  | Products | | | | | | | | | | | |  | |
| --- | --- | --- | --- | --- | --- | --- | --- | --- | --- | --- | --- | --- | --- | --- | --- | --- | --- | --- | --- | --- | --- | --- | --- | --- |
| MFG |  | Protein | Hex | Ac | Lac | For | H_2_ | CO_2_ | H_2_O |  | Ac | Prop | Bu | Lac | Suc | For | H_2_ | CO_2_ | CH_4_ | EtOH | H_2_O | other | |  |
| M1 on hexose^1^ |  |  | 2 |  |  |  |  |  |  |  | 2 | 1 |  |  | 1 |  | 2 | 1 |  |  |  |  | |  |
| M1 on protein^1^ |  | 6 |  |  |  |  |  |  |  |  | 2 | 1 |  |  | 1 |  | 2 | 1 |  |  |  | 1^2^ | |  |
| M2 |  |  | 1 |  |  |  |  |  | 2 |  | 2 |  |  |  |  |  | 4 | 2 |  |  |  |  | |  |
| M3 |  |  | 1 |  |  |  |  |  |  |  | 1 |  |  |  | 1 |  | 1 |  |  |  |  |  | |  |
| M4 |  |  | 6 |  |  |  |  |  | 1 |  | 10 |  |  | 4 |  | 2 |  |  |  | 1 |  |  | |  |
| M5 |  |  | 4 | 2 |  |  |  |  |  |  |  |  | 5 |  |  |  | 6 | 8 |  |  | 2 |  | |  |
| M6 |  |  | 6 | 4 |  |  |  |  |  |  |  |  | 7 | 2 |  | 6 |  | 4 |  |  | 4 |  | |  |
| M7^1^ |  |  | 3 |  |  |  |  |  |  |  | 2 | 4 |  |  |  |  |  | 2 |  |  | 2 |  | |  |
| M7 (on lactate)^1^ |  |  |  |  | 3 |  |  |  |  |  | 1 | 2 |  |  |  |  |  | 1 |  |  | 1 |  | |  |
| M8^1^ |  |  | 10 |  |  |  |  |  | 2 |  | 2 |  | 9 |  |  | 12 | 10 | 8 |  |  |  |  | |  |
| M8 (on Lactate (+Acetate))^1^ |  |  |  | 2 | 4 |  |  |  |  |  |  |  | 3 |  |  |  | 2 | 4 |  |  | 2 |  | |  |
| M9 1 |  |  | 1 |  |  |  |  |  |  |  | 3 |  |  |  |  |  |  |  |  |  |  |  | |  |
| M9 (on CO_2_(+H_2_))^1^ |  |  |  |  |  |  | 4 | 2 |  |  | 1 |  |  |  |  |  |  |  |  |  | 2 |  | |  |
| M9 (on hexose+formate)^1^ |  |  | 1 |  |  | 2 |  |  |  |  | 3 |  |  |  |  |  | 2 | 2 |  |  |  |  | |  |
| M10 (on CO_2_(+H_2_))^1^ |  |  |  |  |  |  | 4 | 1 |  |  |  |  |  |  |  |  |  |  | 1 |  | 2 |  | |  |
| M10 (on formate)^1^ |  |  |  |  |  | 4 |  |  |  |  |  |  |  |  |  |  |  | 3 | 1 |  | 2 |  | |  |

^1^These MFG use several pathways where the pathway yielding the highest growth is preferred (full details in Kettle et al. 2015).

^2^Metabolic products not explicitly listed, assumed to be 50% of total metabolite production from growth on protein

Refs:

Kettle H, Holtrop G, Louis P, Flint HJ. 2018. Micropop: modelling microbial populations and communities in R. *Methods Ecol Evol*. **9**: 399-409.

Kettle H, Louis P, Holtrop G, Duncan SH, Flint HJ. 2015. Modelling the emergent dynamics and major metabolites of the human colonic microbiota. *Environ Microbiol* **17**(5): 1615-30.

1. **Lactate concentration (mM) at which growth of each MFG is assumed to be suppressed by 50%.**

|  |  |  |  | pH 5.5 |  | pH 6.5 |
| --- | --- | --- | --- | --- | --- | --- |
| M1 | Bacteroides | |  | 5 |  | 5 |
| M2 | NoButyStarchDeg | |  | 15 |  | NA^1^ |
| M3 | NoButyFibreDeg | |  | 15 |  | NA |
| M4 | LactateProducers | |  | NA |  | NA |
| M5 | ButyrateProducers1 | |  | 15 |  | NA |
| M6 | ButyrateProducers2 | |  | 15 |  | NA |
| M7 | PropionateProducers | | | 15 or NA^2^ |  | NA |
| M8 | ButyrateProducers3 | |  | 15 or NA^2^ |  | NA |
| M9 | Acetogens | |  | 15 |  | NA |
| M10 | Methanogens | |  | 15 |  | NA |

^1^No suppression on growth

^2^These LUB were modelled assuming either inhibition by lactate or lack of inhibition at pH 5.5 (See Supplementary Figure 5).
